# Unravelling Why and to What Extent the Topology of Similar Ce‐Based MOFs Conditions their Photodynamic: Relevance to Photocatalysis and Photonics

**DOI:** 10.1002/advs.201901020

**Published:** 2019-07-31

**Authors:** Elena Caballero‐Mancebo, Boiko Cohen, Simon Smolders, Dirk E. De Vos, Abderrazzak Douhal

**Affiliations:** ^1^ Departamento de Química Física Facultad de Ciencias Ambientales y Bioquímica, and INAMOL Universidad de Castilla‐La Mancha Avenida Carlos III, S/N 45071 Toledo Spain; ^2^ Centre for Membrane Separations, Adsorption Catalysis and Spectroscopy for Sustainable Solutions Department M^2^S KU Leuven Celestijnenlaan 200F P.O. Box 2454 3001 Leuven Belgium

**Keywords:** excimers, intramolecular charge transfer, ligand metal cluster charge transfer (LCCT), metal–organic frameworks (MOFs), photodynamics

## Abstract

Metal–organic frameworks (MOFs) are emerging materials for luminescent and photochemical applications. Armed with femto to millisecond spectroscopies, and fluorescence microscopy, the photobehaviors of two Ce‐based MOFs are unravelled: Ce‐NU‐1000 and Ce‐CAU‐24‐TBAPy. It is observed that both MOFs show ligand‐to‐cluster charge transfer reactions in ≈100 and ≈70 fs for Ce‐NU‐1000 and Ce‐CAU‐24‐TBAPy, respectively. The formed charge separated states, resulting in electron and hole generation, recombine in different times for each MOF, being longer in Ce‐CAU‐24‐TBAPy: 1.59 and 13.43 µs than in Ce‐NU‐1000: 0.64 and 4.91 µs. The linkers in both MOFs also undergo a very fast intramolecular charge transfer reaction in ≈160 fs. Furthermore, the Ce‐NU‐1000 MOF reveals excimer formation in 50 ps, and lifetime of ≈14 ns. The lack of this interlinkers event in Ce‐CAU‐24‐TBAPy arises from topological restriction and demonstrates the structural differences between the two frameworks. Single‐crystal fluorescence microscopy of Ce‐CAU‐24‐TBAPy shows the presence of a random distribution of defects along the whole crystal, and their impact on the observed photobehavior. These findings reflect the effect of linkers topology and metal clusters orientations on the outcome of electronic excitation of reticular structure, key to their applicability in different fields of science and technology, such as photocatalysis and photonics.

## 1 Introduction

Metal–organic frameworks (MOFs) have been reported as efficient materials for gas storage and separation, catalysis, water cleaning, drug delivery, and bioimaging, to cite some of their applications.^1^, ^2^, ^3^, ^4^, ^5^, ^6^, ^7^, ^8^ In addition to that, their versatile luminescent properties have opened new areas for their application in optoelectronic and sensor devices.^9^, ^10^, ^11^, ^12^, ^13^, ^14^, ^15^, ^16^, ^17^ Therefore, recent advances in experiments and theory have shed light on photoprocesses occurring in MOFs both in suspension and in solid state.^8^, ^18^, ^19^, ^20^, ^21^ For example, for Zr‐based MOFs, studies have elucidated the ultrafast dynamics related to excimer formation, ligand‐to‐cluster charge transfer (LCCT), and energy transfer to encapsulated guests.^18^, ^22^, ^23^, ^24^, ^25^ Other studies have explored the dynamics of Zr‐based MOFs having the same linkers and metal clusters but different crystalline phases, and thus different distances and angles between their neighboring linkers.^26^, ^27^, ^28^, ^29^ These reports have shown the key role of the structural parameters in the interchromophoric interactions that lead to the formation of excimers in a specific network, and to their absence in others even having the same composition, but different structure. From the point of view of theory, MOFs with different metals in the clusters have been examined to understand the relevance of the metal‐linker orbitals interaction.^8^, ^30^ For example, and relevant to the present study, theoretical findings suggest that Ce‐based MOFs may show a better photocatalytic performance than Zr‐based ones.^30^ Ce^4+^ cation has empty and low‐lying 4f orbitals that facilitate the LCCT interaction with the linker's highest occupied molecular orbital (HOMOs). Within the same report, a comparison between different metal clusters suggests that the LCCT has a negative energy, and thus is expected to be spontaneous only for Ce‐MOFs, indicating that the photogenerated charges can be separated through LCCT reactions, increasing the lifetime of these photogenerated states. Electron/hole generation, diffusion, and recombination are key events in the photocatalytic activities and use of MOF‐based LEDs. Thus, it is of great interest to elucidate Ce‐based MOF photochemistry and photophysics, and to explore the relevance of the topology and presence of defects within their reticular structure in the photoinduced processes and outcome.

**Figure 1:**
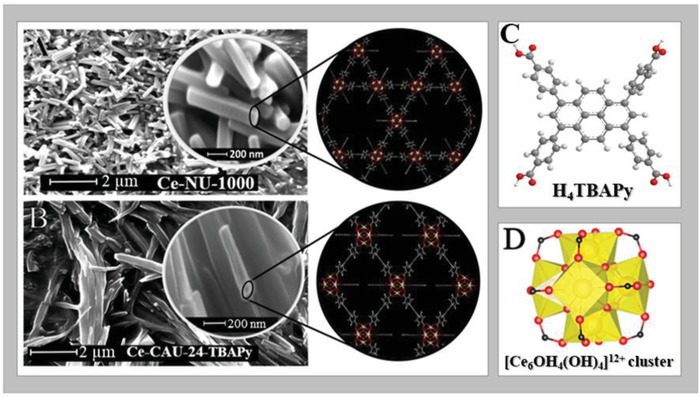
Scanning Electron Microscopy (SEM) images of A) Ce‐NU‐1000 and B) Ce‐CAU‐24‐TBAPy. Structures of C) H_4_TBAPy and D) Ce cluster that form both MOFs.

Herein, we report on femto to millisecond (fs–ms) and single crystal fluorescence studies of two Ce‐based MOFs in acetone suspensions: Ce‐NU‐1000 and Ce‐CAU‐24‐TBAPy (**Figure** 1).^31^ These Ce‐MOFs are not stable in aqueous solutions and, therefore, all experiments were conducted in acetone, which presents similar characteristics to acetonitrile. Many photocatalytic reactions like the reduction of CO_2_ are conducted in acetonitrile and as a result, Ce‐NU‐1000 and Ce‐CAU‐24‐TBAPy are potential photocatalysts for these types of reactions.^32^ Both reticular materials have the same composition, being formed by the 1,3,6,8‐tetrakis(*p*‐benzoate) pyrene (TBAPy^4−^) as linker and hexanuclear cerium oxide clusters. Picosecond resolution experiments indicate that the linker (H_4_TBAPy) molecules in acetone undergo an intramolecular charge transfer (ICT) reaction, which decays in ≈1.6 ns, and a twisting in the excited state of the external rings, which quenches the emission, decaying in ≈90 ps. For the Ce‐NU‐1000 in acetone suspensions, we observed ICT and LCCT reactions of comparable time scale (100–160 fs), and excimer formation (50 ps). The generated structures display times spanning from a few femto to millisecond regime. The structural restrictions in Ce‐CAU‐24‐TBAPy prohibit the excimer formation, while ICT and LCCT events occur in comparable times to the former MOF. The LCCT states show two long‐time relaxation processes (milisecond regime), which are dependently produced by the presence of defects in the crystals, as is clearly supported by time‐resolved single crystal fluorescence microscopy. Ce‐CAU‐24‐TBAPy exhibits a selective photosensitivity to nitroaromatics. Our results clearly show the relevance of topologies and defects in the photobehaviors of two Ce‐based MOFs having similar linkers. The examined photoevents open or close channels on the potential‐energy surfaces leading to very important photoproprieties of MOFs such as those related to photocatalysis, photosensors, and LEDs performance.

## 2 Results and Discussion

### 2.1 Steady‐State Observation

#### 2.1.1 H_4_TBAPy Linker

**Figure 2:**
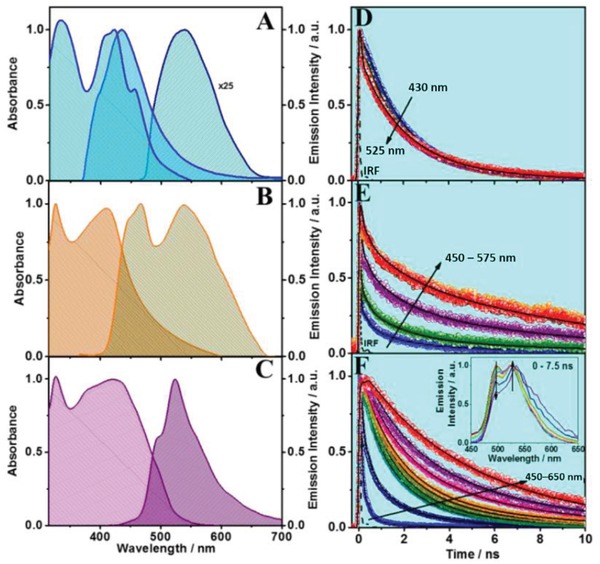
Normalized steady‐state UV–visible absorption and emission spectra (upon excitation at 370 and 450 nm for the second band of the linker) of A) the linker, B) Ce‐NU‐1000 and C) Ce‐CAU‐24‐TBAPy in acetone. Normalized magic‐angle emission decays of D) the linker, E) Ce‐NU‐1000 and F) Ce‐CAU‐24‐TBAPy in acetone upon excitation at 370 nm. The solid black lines are from the best fit using multiexponential functions. The inset in (F) shows the time‐resolved emission spectra of Ce‐CAU‐24‐TBAPy in acetone.

To begin with, **Figure** 2A shows the UV–visible absorption and emission spectra of H_4_TBAPy (the free framework linker) in acetone. The absorption spectrum exhibits two bands: one with the maximum intensity at ≈420 nm, which corresponds to the S_0_ → S_1_ transition observed for pyrene derivatives,^33^ and a second one at ≈330 nm. The latter one can be either due to the absorption of a different species at the ground state (S_0_) or due to S_0_ → S*_n_* (*n* > 1) transitions of the absorbing structures at 420 nm.^33^ Both bands are shifted to longer wavelengths when compared to the spectra of pyrenes lacking the acid groups in H_4_TBpY. The band at ≈420 nm has some vibrational resolution, which is common in molecules with aromatic cores, such as pyrene.^34^ The fluorescence spectrum depends on the excitation wavelength (Figure 2A and Figure S2A, Supporting Information). It presents two bands with intensity maxima at ≈430 and ≈540 nm upon excitation at 370 and 450 nm, respectively. This observation indicates the presence of at least two emitting species of this linker in the acetone solutions. The red shift (5140 cm^−1^) of the green emission band suggests the presence of a process at the electronically first excited state (S_1_). Looking at the molecular structure of H_4_TBpY, this band can be the result of an intramolecular charge‐transfer (ICT) process occurring at S_1_. Figure S2B in the Supporting Information shows that the excitation spectra, gating the signal at both fluorescence bands, do not depend on the emission wavelength, but are very different from the absorption spectrum. This suggests the presence of efficient nonradiative processes upon excitation at the visible side of the absorption band, probably due to ICT events at S_1_ (see later), and upon excitation at the UV‐one, probably due to crossing to higher‐energy nonradiative states. Note that the phenyl carboxylate acid moieties may rotate at S_1_, enhancing the radiationless processes.

#### 2.1.2 Ce‐NU‐1000 MOF

The UV–visible absorption spectrum of Ce‐NU‐1000 in an acetone suspension is also composed by two bands located at ≈325 and ≈410 nm (Figure 2B). The UV‐band is comparable in location and shape to the one of the linker, suggesting that it corresponds to this moiety in the MOF. The second band is broader without vibrational structure, which reflects a stronger interaction of the linkers with the metal clusters. The emission spectrum is clearly composed of two bands with intensity maxima at ≈465 and ≈535 nm. The first one probably originates from the linkers in the MOF, and it is slightly red‐shifted in comparison to that of H_4_TBAPy. We will subsequently explain it in terms of interactions of the linker with the Ce‐oxide clusters in the framework (Figure 1A). The green emission band is much broader, so it might be due to several emissive species. One possibility is excimer formation upon interaction of the linkers at S_1_ and S_0_ states as it has been observed in other MOFs.^18^, ^21^, ^26^, ^29^, ^35^ Examining the structure of Ce‐NU‐1000 MOF (Figure 1A), the linkers forming the triangular holes may be close enough and in a favorable position to produce excimers. The distance (11 Å) and the angle between them (65°) are not ideal for an excimer formation. Nevertheless, comparable situations in other MOFs have been reported, where excimers emission was observed.^18^, ^26^, ^29^, ^35^ Note also that the green emission can be recorded upon excitation at both, the UV and visible absorption bands, which reinforces the explanation involving excimers (Figure S3A, Supporting Information). The excitation spectra gating the green emission do not depend on the observation wavelength, but are different from the absorption one, indicating the involvement of efficient nonradiative events. The fluorescence quantum yield exciting at 370 nm is very low: 10^−4^.

#### 2.1.3 Ce‐CAU‐24‐TBAPy MOF

Figure 2C exhibits the UV–visible absorption and emission spectra of Ce‐CAU‐24‐TBAPy in acetone suspensions. The absorption one shows the same band at ≈325 nm observed in Ce‐NU‐1000, and a broader one with its intensity maximum at ≈420 nm. The broadness suggests the presence of more absorbing species than in Ce‐NU‐1000. However, the emission spectrum consists of a single band with an intensity maximum at ≈525 nm, and two shoulders at ≈500 and ≈575 nm. The difference between this spectrum and that of Ce‐NU‐1000 indicates a different interaction within the MOF structures, reflecting that the reticular topology has an impact on the spectroscopic behavior of this kind of Ce‐MOFs. The absence of the band at ≈540 nm observed in Ce‐Nu‐1000, suggests the lack of or weak emission from excimers. The structure of Ce‐CAU‐24‐TBAPy (Figure 1B) shows small distance variation (12 Å vs 11 Å) but larger angles (72° vs 65°) between the linkers than in Ce‐NU‐1000, which lowers the possibility to form excimers in the former. The emission spectrum does not depend on the excitation wavelength (Figure S4A, Supporting Information), and the excitation spectra (Figure S4B, Supporting Information), independent of the observation wavelength, are different from the absorption spectrum, but similar to those of Ce‐NU‐1000. The fluorescence quantum yield exciting at 370 nm is also low (1%) but higher than that of Ce‐NU‐1000 (Figure S5, Supporting Information).

In this section, we conclude that these Ce‐based MOFs having the same linkers and metal nodes, but a small difference in topology show different emission spectra, in intensity and shape, reflecting the formation (Ce‐NU‐1000) or lack (Ce‐CAU‐24‐TBAPy) of excimers upon photoexcitation. Time‐resolved experiments will give information on the related photoevents.

### 2.2 Picosecond Observation of H_4_TBpY, Ce‐NU‐1000, and Ce‐CAU‐24‐TBAPy

#### 2.2.1 H_4_TBAPy Linker

**Scheme 1:**
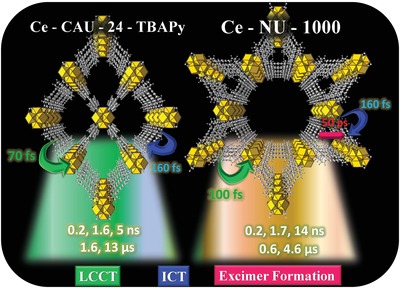
Illustration of the ultrafast processes and their time constants occurring within the excited two Ce‐based MOFs in acetone suspensions: ICT (intramolecular‐charge transfer, blue), LCCT (ligand‐to‐cluster charge transfer, green), and excimer formation (red). The time constants for the emitter to the ground state are indicated at the bottom. More information in Scheme S1.

To explore the photodynamics of these MOFs, we first studied the emission decays of the free linker in acetone. The sample was excited at 370 nm (IRF = 70 ps) and the fluorescence decays were recorded from 430 to 525 nm. Figure 2D shows the results and **Table** 1 gives the obtained data from a multiexponential fit of the decays. The analysis provides three fluorescence lifetimes of 0.09, 1.67, and 3.17 ns that are decaying along the whole observed spectral range. The intermediate one has the highest contribution (*c*_i_) at the bluest part of the spectrum, while the shortest and longest components increase their contributions at longer wavelengths. It has been reported that similar pyrene‐core molecules in solution and having different aromatic substituents, only present single exponential decays of ≈0.5–2.3 ns, corresponding to the monomer emission,^33^ and H_4_TBpY in dimethylformamide shows a single emission of 1.90 ns.^26^ Hence, we assign the intermediate component (1.67 ns) to the emission of the monomer of H_4_TBpY. Previous studies have shown that the pyrene excimers are characterized by very long lifetimes,^36^ so the longest lifetime observed here is probably not of excimers emission, but of a charge‐transfer character species (**Scheme** 1 and Scheme S1A, Supporting Information). Surprisingly, the contribution of the shorter component (≈40 ps) increases at longer wavelengths (Figure 2D and Table 1). We believe that this lifetime is of species that underwent twisting or rotation events of the phenyl carboxylate moieties, enhancing then the nonradiative processes (Scheme 1 and Scheme S1A, Supporting Information).

**Table 1:** Values of the time constants (τ_i_) and pre‐exponential factors (*a*_i_) and contributions (*c*_i_) normalized (to 100) obtained from a multiexponential fit of the emission decays of the indicated samples upon excitation at 370 nm

Sample	λ_Obs/nm_	τ_1_/ns [±0.10]	*a*_1_	*c*_1_	τ_2_/ns [±0.20]	*a*_2_	*c*_2_	τ_3_/ns [±0.20]	*a*_3_	*c*_3_
H_4_TBAPy	430	0.09	24	1	1.67	65	75	3.17	11	24
	475		45	3		44	65		11	32
	525		52	4		30	44		18	52
Ce‐NU‐1000	450	0.18	51	10	1.75	49	90	14.3	–	–
	500		39	1		32	12		29	87
	550		37	1		20	5		43	94
	575		36	1		19	4		45	95
Ce‐CAU‐24‐TBAPy	500	0.19	36	5	1.64	57	64	5.28	7	27
	550		23	2		61	52		16	46
	600		13	1		46	26		41	73
	650		–	–		7	2		93	98

#### 2.2.2 Ce‐NU‐1000 MOF

To elucidate how the topology of neighboring linkers and their interactions with the metal clusters affect Ce‐based MOFs dynamics, we first carried out picosecond experiments on Ce‐NU‐1000 in acetone suspensions. Figure 2E shows the emission decays upon excitation at 370 nm, and Tables 1 and S1 in the Supporting Information give the corresponding parameters obtained from a multiexponential fit. The best fits give emission lifetimes of τ_1_ = 0.18 ns, τ_2_ = 1.75 ns, and τ_3_ = 14.3 ns. The three components decay in the whole observed spectral range. The two shortest ones have their major contribution at short wavelengths, while the longest one starting at 500 nm presents its maximum at longest ones. The second component has a lifetime (1.75 ns) very similar to that of H_4_TBpY in solution (1.67 ns), and therefore it is assigned to the emission lifetime of the linkers in the MOF which do not suffer further photoevents at S_1_. That of the 180 ps is due to monomers strongly interacting with Ce‐clusters, most probably undergoing a ligand‐to‐cluster (metal) charge transfer (LCCT) reaction (Scheme 1 and Scheme S1B, Supporting Information). Finally, the longest component (14.3 ns) is due to excimers emission decay. Comparable lifetimes of excimers of aromatic molecules have been reported.^18^, ^19^, ^35^ However, other MOFs in polymeric matrices, having similar aromatic linkers, but different metal clusters (Fe, In, or Zr) have shown shorter (3–6 ns) emission lifetime of excimers.^26^, ^29^ The optimized structure of Ce‐NU‐1000 using the X‐ray data shows a distance of ≈11 Å and an angle of ≈65° between neighboring linkers (Scheme 1 and Scheme S1B, Supporting Information), which in combination with the different environment created by the metal, may lead to longer excimer lifetimes. The observation of three decaying components is a signature of the heterogeneity of the interactions in the otherwise highly crystalline MOFs, where linker–linker and linker–metal cluster interactions may shape their photobehaviors. Defects have been reported paramount to the performance of an OLED‐based on a Zr‐MOF.^37^ Such differences are also relevant for the photocatalytic use of this kind of materials.

#### 2.2.3 Ce‐CAU‐24‐TBAPy MOF

The topologically different Ce‐CAU‐24‐TBAPy MOF was also studied in acetone suspensions upon excitation at 370 nm. Figure 2F shows the emission decays and Tables 1 and S2 in the Supporting Information give the obtained parameters from the best fit. We got three components of τ_1_ = 0.19 ns, τ_2_ = 1.64 ns, and τ_3_ = 5.28 ns. The contributions of the τ_1_ and τ_2_ components decrease at longer wavelengths of observation, until vanishing in the case of the shortest one, while the contribution of the τ_3_‐component increases. In comparison with the behavior of Ce‐NU‐1000, τ_1_ and τ_2_ are of comparable values. Thus, we assign them to the emissions of the linkers affected and not affected by the metal clusters environments, respectively. The longest component (τ_3_ = 5.28 ns) reflects a different value and behavior (appearing at all observation wavelengths) in comparison with the longest one of Ce‐NU‐1000 (14.3 ns). This remarkable difference in agreement with the steady‐state emission spectra clearly points to the restrictions that presents the framework topology (mainly reflected in different angles between neighboring linkers) of Ce‐CAU‐24‐TBAPy, not allowing excimer formation. Pyrene derivatives with substituents of a high electron density undergo ICT reactions at S_1_. Thus, we suggest that the 5.28 ns component in this MOF is the lifetime of an ICT species (Scheme 1 and Scheme S1C, Supporting Information).

To explore the excess energy effect of the excitation on the outcome of the LCCT and ICT reactions, we performed experiments also exciting Ce‐CAU‐24‐TBAPy at lower energy (430 nm) and compared the contributions of the resulting components in the global emission decays (Figure S7 and Table S3, Supporting Information). The result shows that the contribution of the 0.2 ns component largely decreases upon decreasing the excitation energy. This result is interpreted in terms of localized excitation (within the 320–470 nm range) that induces the LCCT reaction.

To obtain more information on the excited‐state photobehaviors of Ce‐CAU‐24‐TBAPy, we recorded picosecond time‐resolved emission spectra (TRES). The insert of Figure 2F shows the normalized TRES, and Figure S6B in the Supporting Information exhibits the not‐normalized result. First, gating at times shorter than 50 ps, the spectra have their intensity maxima at ≈495 nm. While increasing the gating time, the intensity of this maximum decreases and the green emission band located at ≈525 nm becomes more intense. These two peaks are very similar to those observed in the steady‐state spectrum (Figure 2C). The picosecond ‐resolution of the setup (about 15 ps after a deconvolution of the signal) does not allow to know if the emitting structures are connected or not. However, femtosecond‐experiments will provide a detailed picture (see later). The shoulder in the spectra at longest gating times at ≈570 nm, probably corresponds to species having the lifetime of 5.28 ns, and as a result of an ICT reaction.

As a conclusion of this section, we clearly observed that different topologies of Ce‐based MOFs having identical linkers and metal nodes lead to different photodynamics, reflected in the formation or not of excimers. Defects in these materials might be different in amount, nature, and distribution. The presence of defects and their exact nature has been subject of many investigations in MOFs with M_6_ (M = Zr, Hf, Ce, etc.) clusters and has still not been fully resolved.^38^, ^39^ In Ce‐MOFs, the presence of structural missing‐linker defects has been suggested for Ce‐UiO‐66 but this does not exclude other structural defects like missing clusters.^40^ Thus, we expect that both types can occur in the herein studied Ce‐MOFs. Besides structural defects, it is also likely that small amounts of reduced Ce^3+^ ions are present in the materials. It has been reported that the synthesis solvent can reduce Ce^4+^ of the cluster, ultimately leading to Ce^3+^ containing MOFs or side products.^41^, ^42^ XANES analysis of a Ce_6_‐MOF has shown that up to 1 Ce^3+^ ion per cluster can be present without any loss of structural integrity.^43^ The following time‐resolved single crystal fluorescence microscopy experiments will provide insights on their presence and impact.

### 2.3 Single Crystal Fluorescence Microscopy

**Figure 3:**
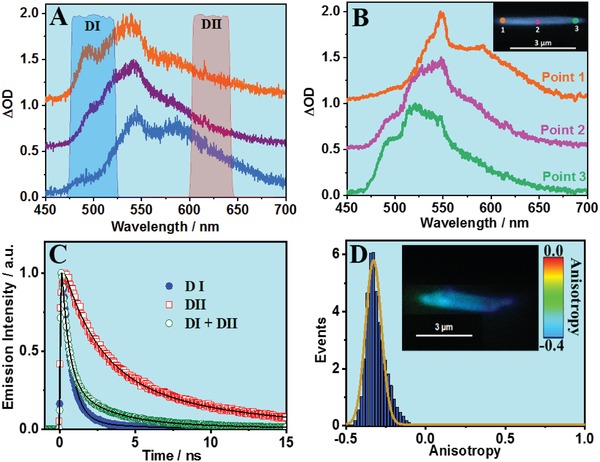
Emission spectra of A) three representative single crystals and B) at three different points in the same single crystal (insert). C) Normalized emission decays collected at two different observation regions as indicated in (A), and D) emission anisotropy distribution of a single crystal, collected under the fluorescence microscope upon excitation at 370 nm.

To this end, we have studied the emission spectra, decays, and anisotropy of single crystals of Ce‐CAU‐24‐TBAPy following picosecond‐excitation at 370 nm. Unfortunately, the emission intensity of the single Ce‐NU‐1000 crystals is very weak and we could not obtain accurate data to analyze. However, we collected the fluorescence lifetime images, spectra, and decays of 20 different Ce‐CAU‐24‐TBAPy crystals and at different points on each individual crystal. **Figure** 3 shows representative emission spectra of different single crystals of Ce‐CAU‐24‐TBAPy (panel A), the spectra collected at selected points on a representative single crystal (panel B), the picosecond emission decays and the analysis at different spectral regions (panel C), and its emission anisotropy (panel D) (Figure S8, Supporting Information). Clearly, the shapes of the emission spectra vary across the different examined single crystals (Figure 3A) indicating a heterogeneous behavior of the emitting species in these crystals. While the maximum of emission intensity is located at the same wavelength (540 nm), the spectra present different shapes along the studied crystals because of the different relative contributions of the emitting species. This result indicates the presence of randomly distributed defects in the MOF framework giving rise to the observed photobehaviors. Remarkably, the heterogeneity is evident also in the spectral behavior within isolated crystals. Figure 3B shows the collected spectra at three different points at a representative single crystal, while the inset gives the fluorescence image of the examined crystal showing the three selected probed zones. Points 1 and 3 are located at the two crystal extremes, while point 2 is located at its center. We did not observe a clear dependence on the interrogated region suggesting a heterogeneous distribution of the defects through the crystal structure. Compared with the steady‐state spectrum (Figure 2C), we observe that this spectrum is an average of those collected under the microscope (spatial resolution: 250–300 nm). Thus, the difference in intensity of each band of the single crystals indicates that the random distribution of defects in the framework gives rise to different emitting species.

To further elucidate the effect of defects on Ce‐CAU‐24‐TBAPy photobehavior, we recoded the emission decays at different points of single crystals. Table S4 in the Supporting Information shows the obtained lifetimes and pre‐exponential factors from the multiexponential fit of the decays (Figure S8, Supporting Information) of the representative crystal (inset of Figure 3B). The fit gives three components of ≈0.5, ≈2.6, and ≈8.5 ns. The values of the time components are longer than those observed in the ensemble average picosecond experiments of the sample in suspension (0.19, 1.64, and 5.28 ns). The difference in the values is explained in terms of the solvent effect on the dynamic process in suspensions. Solvent‐dependent photobehaviors have been recently reported for Zr‐based MOFs,^29^ related to the accessibility of low‐lying polar states in polar solvents. The values of the time components do not vary between the studied crystals and the interrogated points within the same single crystal with the main difference arising from changes in the relative contribution of each component (Table S5, Supporting Information). The shortest component (0.5 ns) has the highest contribution in the global decays collected at point 3 (83%), and it decreases to 69% for the ones recorded at point 1. The opposite behavior is found for the 8.5 ns component. Finally, the contribution of the intermediate component (2.6 ns) is comparable for all the studied points.

To better understand the lifetimes distribution, we studied several crystals collecting their emission decays at two different spectral regions (Figure 3C): from 475 to 525 nm (DI) and from 600 to 650 nm (DII) (Figure 3A). Table S4 in the Supporting Information gives the lifetimes and the pre‐exponential factors (normalized to 100) obtained from the multiexponential fit. The emission collected at DI region decays bi‐exponentially with time constants of 0.53 and 2.40 ns. The relative contribution of the shortest component is 90%, indicating that this is the dominant emitting species in this spectral region. The fit of the decays at DII region gives two components of 2.50 and 8.81 ns. The relative contributions of these two emitters are 60% and 40%, respectively. The larger relative contribution of these two components (40–60%) when we analyze the spectrally resolved emission decays in comparison with the significantly lower one (4–24%) collected without spectral discrimination indicates that the main emitters for the whole crystal show the shortest component (0.5 ns).

Finally, to obtain information on the orientation of the emitting structures we studied the emission anisotropic behavior of several Ce‐CAU‐24‐TBAPy single crystals. Figure 3D shows a representative image of a crystal, and a representative histogram of the steady‐state anisotropy value distribution in the same crystal. Additional examples are given in Figure S9 in the Supporting Information. The histogram is fitted by a Gaussian function which shows a distribution with maximum at −0.33 (full‐width at half maximum of intensity, FWHM = 0.10). This behavior and the value of the steady‐state anisotropy indicate an ordered periodic structure and preferential orientation of the dipole moment of the emitters within the single crystal, in agreement with the high crystallinity if this MOF (Figure 1 and Figure S1, Supporting Information). It should be noted that the fluorescence lifetime imaging and the anisotropy value distribution (under our experimental conditions, limited by a spatial resolution of 250–300 nm) along the studied crystals cannot provide correct description of the defects: type, number, and localization in the crystals.

### 2.4 Nanosecond–Microsecond Transient Absorption Observations

To obtain information on the slow relaxation pathways from the excited states of these MOFs in acetone, we performed flash photolysis transient absorption (TA) experiments (nanosecond–microsecond time scale). Figures S10 and S11 in the Supporting Information show the transient absorption spectra (TAS) upon excitation at 430 nm and gating at different times, and the corresponding transient decays at selected wavelengths of probing. Table S6 in the Supporting Information exhibits the parameters obtained from the fit of the decays. We first discuss the behavior of the free linker in acetone. The intensities of its TAS are very low at any gating time (Figure S10A, Supporting Information). After a long‐time accumulation, we were able to collect its TA decays at 450, 500, and 600 nm (Figure S10D, Supporting Information). The three transients are due to two long nonemissive species of 0.56 and 2.15 µs. When the solution is saturated with O_2_ or N_2_, the time constants do not vary (Figure S12A, Supporting Information), indicating that they do not originate from triplet states. Exciting at 355 nm gives the same result, indicating that we are interrogating the same species of the linker (Figure S13, Supporting Information). We suggest that these two absorbing species correspond to twisted and ICT intermediates between the singlet and fundamental states of the species described in the picosecond section.

We then analyzed the behavior of both MOFs in acetone suspensions. For Ce‐NU‐1000, the TAS show a relatively strong continuous negative band in the probed visible region (450–750 nm) with the maximum of intensity at 720 nm (Figure S10B, Supporting Information). Similar TAS, showing a continuous signal, is shown by Ce‐CAU‐24‐TBAPy (Figure S10C, Supporting Information). This kind of continuous spectra have been already recorded for other MOFs,^44^, ^45^, ^46^, ^47^ and the behaviors were attributed to long‐lived charge‐separated states (CSSs) in these materials. The incorporation of Ce^4+^ metal nodes in hybrid materials has been suggested to facilitate LCCT reaction.^8^, ^30^ The empty 4f orbitals of Ce^4+^ are found distributed within the gap of the linker orbitals, allowing a favorable LCCT event, which is desirable to obtain long‐lived excited states, and significantly increasing the photocatalytic performance of these materials.^30^ The fits of the transient signal give two components of 0.64 and 4.91 µs for Ce‐NU‐1000, and 1.59 and 13.43 µs for Ce‐CAU‐24‐TBAPy, which are attributed to the time constants of e^−^–h^+^ pair recombination. Other MOFs, behaving as semiconductors, have shown multiexponential e^−^–h^+^ recombination, attributed to shallow and deeper trapped states.^18^, ^48^ It has been also suggested that due to the complexity of the metal cluster particles and presence of defects, different trapped states can be formed, producing different e^−^–h^+^ recombination times. Thus, based on previous reports,^18^, ^48^ we suggest that the shortest decaying component (0.64 and 1.59 µs) corresponds to the e^−^–h^+^ recombination from a shallow trap states, while the longest one (4.91 and 13.43 µs) comes from deeper ones. It is worth to note that while having the same linkers and Ce‐based metal clusters, the different topologies of these MOFs also lead to different e^−^–h^+^ recombination times, which are three times longer for Ce‐CAU‐24‐TBAPy than for Ce‐NU‐1000. Thus, not only the neighboring linkers arrangement (distance and angle) is key in the photobehaviors of these MOFs, but also the metal clusters environment and orientation affect the long time relaxation processes, relevant to the performance of MOFs in photocatalysis, and OLED.^37^, ^49^ Although the mechanisms of photosensing (dynamic quenching) and photocatalysis are intrinsically different, they both depend on the diffusion and collision of the involved reactants. Therefore, the presence of long‐lived charge‐separated excited states in the two Ce‐MOFs can enhance their photocatalytic activity or increase their efficiency as photosensors by decreasing the probability that the photogenerated electrons and holes recombine before accessing adsorbed reactants.^50^, ^51^ This is especially valid for Ce‐CAU‐24‐TBAPy, where the charge separated states live longer (1.59 and 13.43 µs). It should be noted that, while we cannot determine if the number and nature of defects are different in these Ce‐based MOFs, there is a small difference in the metal‐clusters orientation in both MOFs: the metal clusters in Ce‐CAU‐24‐TBAPy are all oriented in the same direction, which is not the case for Ce‐NU‐1000 with 3 different orientations. As this situation produces different topologies of this Ce‐based MOF, it could lead to different (in nature and number) defects on the metal clusters (Figure 1A,B), and therefore different e^−^–h^+^ recombination times in the deeper and shallow trap states.

The generation of electrons and holes in MOFs, reminiscent to the photobehavior of semiconductors, has been demonstrated by analyzing their reductive and oxidizing power when interacting with relevant organic compounds. *N*,*N*,*N*′,*N*′‐tetramethyl‐phenylenediamine (TMPD) has been used to evaluate the reductive and oxidizing ability of MOFs.^18^, ^46^, ^52^ The feasibility of Ce‐MOFs as photocatalyst has recently been investigated theoretically by calculating the density of states for several Ce‐MOFs and the authors have suggested that the UiO‐66(Ce)‐NH_2_ MOF would be able to photocatalyze CO_2_ reduction.^30^ The radical monocation structure (TMPD^+^) is easily detected, presenting a characteristic absorption spectrum with two intensity maxima at ≈550 and ≈600 nm. Figure S14 in the Supporting Information shows the appearance of the TMPD^+^ band in both MOFs/TMPD suspensions even without any exposure to laser excitation. This fact indicates an electronic interaction with MOF, generating TMPD^+^ species at the ground state. A plausible explanation is that the Ce‐MOFs already have a partial charge‐transfer character at S_0_ where a charge has migrated from the pyrene linkers to the Ce‐clusters. In this scenario and following the observation, the HOMO of TMPD should be higher than that of the delocalized charge‐transfer molecular orbital of the MOFs. In any scenario, our observation shows that Ce‐based MOFs are suitable materials for catalysis as they already present LCCT character at the ground state in agreement with the theoretical prediction.^30^ Thus, improving this ability could be of great importance to catalysis using MOFs without light.

Summarizing this section, both MOFs photoproduce long‐living CSSs as a results of LCCT reaction, being the e^−^/h^+^ recombination times almost three times longer in Ce‐CAU‐24‐TBAPy MOF than in Ce‐Nu‐1000 one, probably reflecting different defects on the metal clusters. Interestingly, even in the absence of light irradiation, both MOFs give an electron to the well‐known molecular probe, TMPD, opening the way to its possible use in catalysis without light.

### 2.5 Femtosecond Time‐Resolved Experiments

In the previous section, we have invoked and discussed the presence of LCCT and ICT reactions. However, we could not give the time scale of these events, due to the longer time‐resolution of the picosecond‐system. Below, we discuss results of experiments using femtosecond‐resolution of both fluorescence up‐conversion and UV‐visible‐NIR TA spectroscopies aiming to elucidate both ultrafast dynamics and spectroscopy of the samples studied here.

#### 2.5.1 Femtosecond Time‐Resolved Up‐Conversion Emission Observation

To explore the ultrafast processes that occur in the excited state of the MOFs, we carried out femtosecond experiments in acetone suspensions. We excited at 370 nm and gated the emission at different wavelengths. The very low quantum yield of Ce‐NU‐1000 requires very long collecting times in the femtosecond‐experiments, which lead to a photodegradation of the sample, and therefore the impossibility to carry out the experiments under a high repetition rate (80 MHz). However, this barrier was overcome using femtosecond‐TA spectroscopy working at lower repetition rate (1 kHz). The data are shown in Section 2.5.2.

On the other hand, femtosecond‐emission experiments on Ce‐CAU‐24‐TBAPy in acetone were performed. Figure S15 in the Supporting Information displays the recorded transients in a short time window, while Figure S16 in the Supporting Information shows them in a longer one. Table S7 in the Supporting Information exhibits the values of the time constants and the pre‐exponential factors (normalized to 100) using multiexponential fits. We obtained two components of ≈160 fs and ≈225 ps. The longest component has a time similar to the shortest one obtained in the picosecond experiments, assigned to the linker species interacting with Ce‐clusters. The presence of a femtosecond‐component that decays from 440 to 530 nm and rises from 550 nm to the end of the probed spectral range indicates the presence of an ultrafast reaction at S_1_. Based on the spectral and dynamical behavior of this component, we assigned it to an ICT reaction happening within few of the excited linkers (Scheme S1C, Supporting Information). Note that in this short time regime, intramolecular vibrational‐energy redistribution (IVR, 100s of fs) and vibrational relaxation (VR, few picoseconds) processes are also taking place, and therefore it is possible that the observed 160 fs is a combination of the ICT component with these events. LCCT reaction should be also very fast, but it generates nonfluorescent species at the studied spectral region and thus the rising component cannot include this process. However, this will be examined in the following section.

#### 2.5.2 Femtosecond Time‐Resolved UV–Visible–NIR Transient Absorption Observation

**Figure 4:**
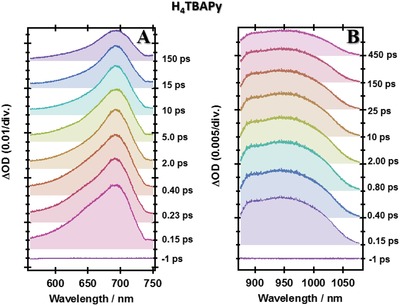
Time evolution of the transient absorption spectrum (TAS) of H_4_TBAPy in acetone at A) visible and B) NIR spectral regions. ΔOD is the change in the optical density upon excitation at 400 nm and recording at different pump‐probe delays.

**Figure 5:**
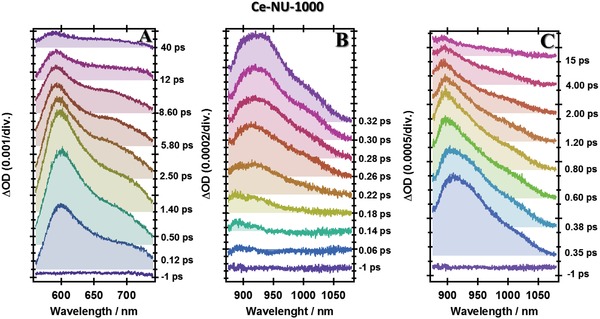
Time evolution of the transient absorption spectrum (TAS) of Ce‐NU‐1000 in acetone at A) visible, B,C) NIR spectral regions, at short and long gating times, respectively. ΔOD is the change in the optical density upon excitation at 400 nm and recording at different pump‐probe delays.

**Figure 6:**
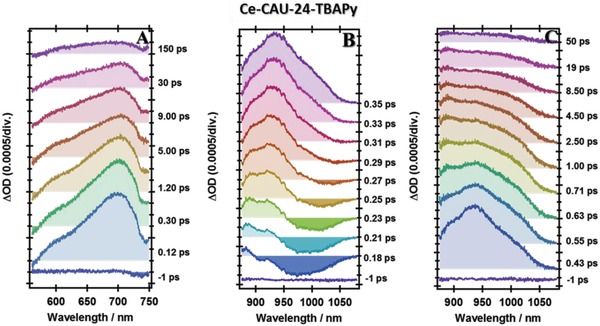
Time evolution of the transient absorption spectrum (TAS) of Ce‐CAU‐24‐TBAPy in acetone at A) visible, B,C) NIR spectral regions, at short and long gating times, respectively. ΔOD is the change in the optical density upon excitation at 400 nm and recording at different pump‐probe delays.

To get a full understanding of the photobehaviors of these MOFs, we have carried out femtosecond‐TA experiments. The ultrafast electronic (400 nm, ≈100 fs excitation, 1 kHz) excitation brings the system to the S_1_ potential‐energy surface where the photoevents start taking place to direct the wavepacket to other wells on the potential‐energy surfaces. **Figures** 4, 5 and 6 show the visible–NIR transient absorption spectra of H_4_TBAPy, Ce‐NU‐1000, and Ce‐CAU‐24‐TBAPy, respectively. Due to the low solubility of the free linker in acetone, we used a 1:1 acetone:DMF solution to increase its solubility. However, both MOFs were measured in acetone suspensions. The visible TAS of the linker show the time evolution of a band located at ≈700 nm (Figure 4A). This band exhibits a shoulder at shorter probed wavelengths (≈650 nm) which seems to disappear in ≈2 ps. On the other hand, the NIR‐TAS show a broad positive band with two intensity maxima centered at ≈900 and ≈980 nm (Figure 4B). Both maxima are formed in less than 120 fs.

Ce‐NU‐1000 TAS display an intense band at ≈600 nm. This band is formed in less than 120 fs and reaches its maximum in ≈1 ps (Figure 5A). A weaker band is recorded in the 700 nm region and starts decreasing while showing a shift to lower wavelengths. For clarity, the NIR‐TAS are presented in panels B and C of Figure 5. Panel B and Figure S17 in the Supporting Information show a negative band centered at ≈1000 nm, and which disappears in ≈150 fs. It is worthy to notice that the very early negative signal in this band does not appear in the NIR‐TAS of the free linker (Figure 4B). The NIR spectra of Ce‐NU‐1000 originate from a positive absorption band centered at ≈925 nm. When the gating time increases, a shoulder appears at ≈1000 nm. Note also the shift of the 925 nm band to lower absorption wavelengths at times longer than ≈320 fs (Figure 5C).

Clearly, the spectral features at both, visible and NIR‐TAS of the MOF, are different from those of the free linker, reflecting the occurrence of different femtosecond‐events in the reticular structure. To begin with, the early and negative signal at ≈1000 nm in the MOF's TAS at the NIR region, is a clear evidence of a LCCT reaction which cannot happen in the free linker. The negative band is an instantaneous emission from a charge‐separated state, ultrafast generated (≈100 fs) due to LCCT reaction, and which has not yet relaxed to other states as those of deeper and shallow ones invoked above in the flash photolysis observation. The ultrafast LCCT event involves the strong overlap of the available 4f orbitals of the Ce‐clusters with those of the linker orbitals gap. Further relaxation leads to the formation of species absorbing in the visible–NIR spectral region.

Now, we examine the TAS of Ce‐CAU‐24‐TBAPy (Figure 6). To begin with the visible region, an instantaneous positive band appears at times shorter than 120 fs, having its intensity maximum at 700 nm, and a shoulder at ≈600 nm. These spectral features are reminiscent to those of the free linker (Figure 4A) but are very different from those of Ce‐NU‐1000 MOF (Figure 5A). However, and it is worthy to remark, that the NIR‐TAS of Ce‐CAU‐24‐TBAPy are very different from those of Ce‐NU‐1000, except for the stronger negative signal at ≈1000 nm (Figure 6C).

Figure S18 in the Supporting Information shows a comparative illustration of TAS at different regions and delay times of the probe. Clearly, the MOFs behave differently, reflecting different formation and yield of formed transient species. In agreement with the femtosecond‐emission experiments (Figures S15 and S16, and Table S7, Supporting Information), the rising TAS (in terms of increase in the transient optical density) at the visible region are due to the absorption of ICT species. Thus, the positive bands in the 650–750 nm region in both MOFs mainly originate from the ICT structures of the linkers in the MOFs. Further examination of the dynamical behavior of these bands suggests a rising component of ≈50 ps in the case of Ce‐NU‐1000, assigned to excimer absorption in agreement with the steady‐state emission spectrum (Figure 2B). The TA band of Ce‐NU‐1000 at 600 nm clearly reflects the formation of LCCT species as it does not appear in the H_4_TBAPy TAS. However, for Ce‐CAU‐24‐TBAPy, this band is not recorded, most probably due to the strong emission of this MOF in this region, contrary to the weaker one of Ce‐NU‐1000 (Figure 2).

Now, we compare and assign the spectral features in the NIR region. To begin with very short time observations (150 fs, Figure S18, Supporting Information), the positive band of H_4_TBAPy is the absorption of ICT species as it happens in the visible one. However, the negative bands of both MOFs, being stronger in the case of Ce‐CAU‐24‐TBAPy, are due to emission from instantaneously (within the excitation pulse, ≈100 fs) created LCCT structures within the frameworks. The positive band appearing at ≈880 nm also reflects the formation of these species, leading to “semiconductor‐like” materials where electrons and holes (e^−^/h^+^) are created, separated, diffused, and later recombined to the ground state.^18^ We already discussed the recombination process in the section of flash photolysis. At longer time delays, the intensity of these bands becomes stronger (Figure S18C, Supporting Information). The relative intensities of the positive bands at ≈1000 nm, assigned to ICT structures of the linkers in both MOFs, suggest that these species are less efficiently formed in the Ce‐NU‐1000 framework, in agreement with the low absorption intensity at ≈700 nm.

After identifying the transient absorption and emission bands, we proceed now to analyze their femtosecond‐dynamics. Figure S19 in the Supporting Information shows representative decays at selected probing wavelengths according to the previous spectral assignment. Figure S20 in the Supporting Information shows the decays at other probing wavelengths in the NIR region. The data from the multiexponential fits of these transients are given in **Table** 2. To begin with the visible region, the transients of the three samples exhibit a very fast rising component of 100–200 fs. For H_4_TBAPy, this component is due to ICT, as we discussed in the femtosecond‐TAS section. For both MOFs, it embraces ICT and LCCT reactions, noting that Ce‐CAU‐24‐TBAPy exhibits the fastest one (90–150 fs). Ce‐NU‐1000 also shows a 50 ps rising component at 700 nm, due to the formation of excimers (Figure S19B, Supporting Information). In addition to these rising signals, the fits also give 3–7 ps components due to VR/cooling in the excited systems.

**Table 2:** Values of the time constants and normalized (to 100) preexponential factors (*a*_i_) obtained from the best fit of the transients of Ce‐CAU‐24‐TBAPy, Ce‐NU‐1000 and linker upon excitation at 400 nm and observation at the indicated wavelengths

λ_Obs_/nm	Sample	τ_1_/ps [±0.05]	*a*_1_	τ_2_/ps [±0.5]	*a*_2_	τ_3_/ps [±0.5]	*a*_3_	τ_4_/psa)	*a*_4_
600	Ce‐CAU‐24‐TBAPy	0.15	−100			6.32	46	450	54
	Ce‐NU‐1000	0.21	−100			6.87	67	450	33
	H_4_TBAPy	0.15	−100			5.91	30	1000	70
700	Ce‐CAU‐24‐TBAPy	0.09	−100	3.01	51			450	49
	Ce‐NU‐1000	0.12	−100	5.41	59	51	−23	450	41
	H_4_TBAPy	0.16	−100	3.72	23			1000	64
880	Ce‐CAU‐24‐TBAPy	0.23	−100	5.51	64			450	36
	Ce‐NU‐1000	0.15	−100	5.13	65			450	35
900	Ce‐CAU‐24‐TBAPy	0.30	−100	3.81	67			450	33
	Ce‐NU‐1000	0.13	−100	3.17	66			450	34
	H_4_TBAPy	0.52	−61	2.35	37	5.37	−39	1000	63
940	Ce‐CAU‐24‐TBAPyb)	0.25	9	5.57	58			450	33
	Ce‐NU‐1000	0.35	51	5.13	37			450	12
	H_4_TBAPy	0.52	−61	2.35	37	5.37	−39	1000	63
980	Ce‐CAU‐24‐TBAPy	0.07	−53c)	0.08	−47	4.11	31	450	69
	Ce‐NU‐1000	0.10	−44c)	0.13	−56	2.42	35	450	65

a) Fixed values from the ones obtained in the picosecond experiments

b) A very short (<70 fs) and weak negative signal is recorded at this wavelength (Figure S18, Supporting Information)

c) Corresponds to the decay of the negative signal.

To further elucidate the femtosecond‐dynamics, we analyzed the transients in the NIR region where we observed ultrafast decaying and rising components sharing a common channel. H_4_TBAPy transients (at 900 and 940 nm) exhibit a rising component of 520 fs due to ICT and ultrafast solvation (acetone is a polar solvent) of the formed species (Table 2). The fit also gives another rising component (5.4 ps), most probably due to twisting of the phenyl carboxylate groups (Figure 1C), and a decaying one (2.4 ps) reflecting VR/cooling. As we already noted above, the fs‐TAS of both MOFs show very short living emitting structures around 980 nm, assigned to emitting LCCT species (Figures S17 and S18, Supporting Information). The lifetime of these emitters is very short: ≈70–100 fs (Table 2). We got slightly longer times (130–350 fs) when observing the rising positive TA band at 880 or 990 nm and decaying one at 940 nm (Figures S18–S20, Supporting Information). These relatively longer rising times, as well as for the linker solution, are probably affected by the ultrafast solvation (femtosecond‐regime) of acetone. In addition to these components assigned to ICT and LCCT events, we also got components of 3–6 ps due to VR/cooling events. A previous study on Zr‐pyrene based MOFs (NU‐1000 and NU‐901) did not observe the formation of LCCT species within the framework.^37^ The results were interpreted in terms of interchromophoric interactions, leading to excimer formation (2 ps) in the case of NU‐901 and invoking polar excited states in the case of NU‐1000. Comparing the femtosecond‐behavior of these MOFs with the studied here, the presence of Ce‐clusters in the network allows the production (≈100 fs) of LCCT structures, most probably due to the interaction of the 4f orbitals of Ce with those of the pyrene units. Theoretical studies of the effect of metal (Zr, Hf, Th, Ti, U, and Ce) on the LCCT formation in UiO‐66(Ce) MOF have suggested the production of this kind of Ce‐ligand interactions.^30^ Our experimental results are in full agreement with the theoretical prediction, and are the first ones to experimentally elucidate such dynamics for two Ce‐based MOFs. It is of great interest to the MOF's community and growing up subfield of photonics of MOFs to carry out advanced spectroscopic studies of this kind of reticular structures, changing the metal clusters, but keeping the same linker moiety, exploring the fs–ms dynamics under the same experimental conditions (solvent, pulse width, excitation fluence, etc). This will provide more clues to a better design of these materials for photonic applications. Table S8 in the Supporting Information summarizes the above discussion where we indicate the main absorbing structures, and the produced events in the interrogated spectral regions.

To summarize the fs‐observations using both, up‐conversion and transient‐absorption techniques, for both MOFs in acetone suspensions we observed ≈100 fs decaying and rising components due to ICT and LCCT reactions. These times become slightly longer (up to 350 fs) at the NIR interrogated region, probably due to ultrafast solvation. A 2.5–7 ps decaying component is recorded because of VR/cooling. Furthermore, Ce‐NU‐1000, contrary to Ce‐CAU‐24‐TBAPy, shows excimer formation in 50 ps. Remarkably, the very early (less than 100 fs) recorded negative TAS of the MOFs at the NIR region, absent in the free linker, clearly reflecting the ultrafast formation of LCCT structures in the reticular porous materials. These evolve to longer living (µs scale) absorbing and e^−^/h^+^ emitting species, in agreement with the µs‐flash photolysis experiments. The photoconversion and recombination processes are reminiscent to the behavior of semiconductors. Schemes 1 and S1, and Table S8 in the Supporting Information illustrate the fs–µs dynamics observed and discussed in the three examined samples in acetone.

### 2.6 Ce‐CAU‐24‐TBAPy as Photosensor for Nitroaromatic Explosives

Luminescent MOFs have been proposed as fluorescent sensors in several fields of science and technology.^10^ For example, they have been reported as photosensors of nitroaromatic molecules, showing a strong quenching of their fluorescence when interacting with them.^10^, ^11^, ^53^, ^54^, ^55^ It has been shown that the driving force behind quenching mechanism is the result of the formation of a charge‐transfer complex where in some cases H‐bonds between the MOFs and the nitroaromatics play a key role in the selectivity and sensitivity to detect these compounds. Based on the above findings, we explored the ability of the present Ce‐based MOFs to detect nitroaromatics in acetone solution. Because Ce‐NU‐1000 has a very low fluorescence quantum yield (<10^−4^), we did not examine it. However, we explored the ability of Ce‐CAU‐24‐TBAPy in acetone to detect small quantities (up to 500 ppm in 3 mL of solution) of nitroaromatic molecules. Figures S21–S24 in the Supporting Information show its emission response when interacting with trinitrophenol (TNP), 1, 3, 5‐trinitrobenzene (TNB), 3‐nitrotoluene (NT), and nitrobenzene (NB), respectively. In all the samples, the emission spectra exhibit a clear quenching. To get information on the nature of the mechanism (dynamic, static or a combination of both), we analyzed the steady‐state and time‐resolved data. Figures S21–S24 and Table S9 in the Supporting Information show the experimental results and their analysis. For TNP, NT and NB aromatics, the Stern–Volmer analysis gives a straight plot, suggesting a static quenching.^56^

However, for TNB detection the resulting nonlinear behavior indicates a dynamical quenching mechanism (or a combination with the static one). Based on the chemical structure of Ce‐CAU‐24‐TBAPy, we exclude formation of H‐bonds with the nitroaromatics, as it lacks groups able to exhibit such interactions. We believe that the emission quenching rather involves a charge‐transfer process. Using suspension of Ce‐CAU‐24‐TBAPy in acetone having the same concentration of MOF (same optical density), and adding 500 ppm of the nitroaromatics, we found the following ratio of the emission quenching ($\frac{I_{0}-I}{I_{0}}\times 100$ at 525 nm, *I*_0_ and *I* are the emission intensities without and with the added amount of the nitroaromatics): NT (47%) > NB (45%) > TNB (41%) > TNP (36%). The Stern–Volmer associated constants (*K*_SV_) for the case of a static quenching are 2.31 × 10^2^ M^−1^ (NT), 1.99 × 10^2^ M^−1^ (NB), and 2.26 × 10^2^ M^−1^ (TNP). These values are not high enough to propose Ce‐CAU‐24‐TBAPy as new photosensor of nitroaromatics. However, we found that this MOF is not sensitive at all to toluene, benzylic alcohol and p‐aminophenol (Figure S25, Supporting Information). Thus, even though it does not show a high sensitivity to nitroaromatics, it is highly selective toward these compounds.

## 3 Conclusion

In this work, armed with the potential of fast and ultrafast spectroscopies, we have shown and discussed the photobehavior (absorption and emission spectra, fs–ms dynamics and lifetimes) of two Ce‐based MOFs having pyrene as the main part of the linker moiety: Ce‐NU‐1000 and Ce‐CAU‐24‐TBAPy. Both MOFs, having the same metal clusters and linkers, show however different spectroscopies and dynamics, unraveling the topological difference. For example, while the Ce‐NU‐1000 presents excimers formation (in 50 ps and lifetime of 14.3 ns) after suffering an ICT reaction, Ce‐CAU‐24‐TBAPy only shows the ICT event. The lack of excimers formation in the later is the result of structural restrictions not allowing mutual interactions between neighboring organic linkers in its framework. The ICT states within the organic linkers of both MOFs are, however, formed in comparable times (≈160 fs). The interaction of the available 4f orbitals of Ce^4+^ atoms with ones of the HOMO linkers results in a very fast LCCT reaction: ≈70 fs in Ce‐CAU‐24‐TBAPy, and ≈100 fs in Ce‐NU‐1000. It is worthy to notice that these behaviors are different from those observed in Zr‐NU‐1000 and Zr‐NU‐901 MOFs, having the same linkers but different metal clusters. Of relevance to the use of MOFs in photoscience and related technologies, we found that the photoproduced LCCT structures, having separated electron and hole that recombine in two different times, reflecting the presence of shallow (short lifetime) and deeper (long one) trap states on the metal clusters network. The e^−^/h^+^ recombination is almost three times longer in Ce‐CAU‐24‐TBAPy (1.6 and 13.4 µs) than in Ce‐Nu‐1000 (0.6 and 4.9 µs). We suggest that the difference could be related to the different metal‐clusters orientations in both MOFs, being all oriented in the same direction within the former, but of 3 different orientations within the later. Single crystals fluorescence microscopy of Ce‐CAU‐24‐TBAPy reveals a heterogenous presence of defects within its framework, as we recorded different emission spectra of several single crystals, and at different locations on the same individual crystal. The emission anisotropy analysis of single crystals showed an ordered structure with preferential dipole moment orientation of the emitters. Finally, we also found that Ce‐CAU‐24‐TBAPy has a potential and selective sensitivity to nitroaromatics. The presented findings provide direct information on the clues that shape the photobehaviors and outcome of two chemically similar but topologically different Ce‐based MOFs, at very short and longtime scales. We believe that the results are of high quality that allow a deep understanding of the photobehavior of these materials. Thus, this will form a solid base for future developments in photocatalysis and optoelectronics using these materials.

## 4 Experimental Section

*Synthesis of the Materials—Molecular Ce‐clusters*: [Ce_6_O_4_(OH)_4_(NH_3_CH_2_COO)_8_(NO_3_)_4_(H_2_O)_6_Cl_8_·8H_2_O] clusters were synthesized according to the literature.^57^ First, 9 g of glycine (120 mmol) and 30 g of cerium ammonium nitrate (CAN; 55 mmol) were dissolved in 27 g of water. To this, 321 g of a saturated NaCl solution was added and the resulting solution was left at room temperature for 48 h. The precipitated crystals were filtered off and dried at 60 °C.

Ce‐CAU‐24‐TBAPy MOF was synthesized according to literature.^31^ First, 10 mg of 1,3,6,8‐tetrakis (*p*‐benzoic acid) pyrene (H_4_TBAPy; 16 µmol) and 675 mg of benzoic acid (5.5 mmol) were dissolved in 1.2 mL of *N*,*N′*‐diethylformamide (DEF) and heated to 100 °C. To this, 23.5 mg of molecular Ce clusters (60 µmol Ce) in 250 µL of water were added and the resulting mixture was kept at 100 °C under stirring for 15 min. Afterward, the precipitate was isolated by centrifugation.

Ce‐NU‐1000 was synthesized similar to literature reports.^31^ First, 10 mg of H_4_TBAPy (16 µmol) and 675 mg of benzoic acid (5.5 mmol) were dissolved in 2 mL of DMF and heated to 100 °C. To this, 23.5 mg of molecular Ce clusters (60 µmol Ce) in 250 µL of water were added and the resulting mixture was kept at 100 °C under stirring for 15 min. Afterward, the precipitate was isolated by centrifugation.

*Structural, Spectroscopic, and Dynamic Measurements*: The characterization of the materials was carried out by Powder X‐ray Diffraction (PXRD), recorded on a Malvern PANalytical Empyrean diffractometer in transmission mode using a PIXcel3D detector and a Cu anode (Cu Kα1: 1.5406 Å; Cu Kα2: 1.5444 Å) source. Both materials are very crystalline, showing a well resolved PXRD pattern (Figure S1, Supporting Information). Scanning electron microscopy (SEM) images were also recorded, using a Philips XL30 FEG microscope. Samples were coated with 2 nm Pt prior to imaging (Figure 1). The Ce‐NU‐1000 particles are hexagonal rods with a length of 600–900 nm and a diameter of 100–200 nm. The particles of Ce‐CAU‐24‐TBAPy are less well‐defined and rod‐shaped with a length and diameter of 0.4–3 µm and 150–500 nm, respectively (Figure 1).

Below, we briefly describe the spectroscopic techniques used for the study of the materials (see Supporting Information for more information). The steady‐state UV–visible absorption and fluorescence spectra were recorded using JASCO V‐670 and FluoroMax‐4 (Jobin‐Yvon) spectrophotometers, respectively. The picosecond (ps) time‐resolved emission experiments have been carried out employing a ps time‐correlated single‐photon counting (TSCPC) system.^58^ The samples were excited at 370 nm using a 40 ps‐pulsed diode laser (≈1 mW, 40 MHz). The instrument response function (IRF) of the set‐up is ≈70 ps. The confocal fluorescence microscopy measurements were performed on a MicroTime 200 confocal microscope (PicoQuant). The excitation was conducted with the same diode laser used in the TSCPC experiments. The emission spectra were collected through a Shamrock ST‐303i (Andor Technology) imaging spectrograph and detected using an Andor Newton EMCCD camera (Andor Technology). The samples were measured in the solid state and were prepared by depositing few crystals on the coverslip and introducing it into the sample holder. The nanosecond (ns) flash photolysis setup has been described previously.^59^ To excite the sample, the laser beam was used from an optical parametric oscillator (OPO) (pumped by a Q‐switched Nd:YAG laser, Brilliant, Quantel) at 430 nm. For probing, we use the output of a 150 W Xenon arc lamp. The measured IRF of the system is ≈8 ns.

The femtosecond (fs) UV–visible–NIR transient absorption experiments were done using a chirped pulse amplification setup.^60^ The samples were placed in a 1 mm spinning cell, excited at 400 nm (≈350 µW) and probed from 450 to 1100 nm (1 kHz repetition rate). The IRF, measured in terms of ΔOD from Raman scattering of pure acetone, was 120 fs. Femtosecond time‐resolved emission transients were collected using the fluorescence up‐conversion technique.^61^ The sample was excited at 370 nm (≈5–10 mW) and the emission was gated at different wavelengths using the 800 nm probing beam. The IRF of the apparatus (measured as a Raman signal of pure solvent) was ≈270 fs (full width at half‐maximum, FWHM) at the excitation wavelength.

## Conflict of Interest

The authors declare no conflict of interest.

## Supporting information

Supplementary

## References

[1] M. J. Kalmutzki , N. Hanikel , O. M. Yaghi , Sci. Adv. 2018, 4, eaat9180. 30310868 10.1126/sciadv.aat9180 PMC6173525

[2] A. Schoedel , M. Li , D. Li , M. O'Keeffe , O. M. Yaghi , Chem. Rev. 2016, 116, 12466. 27627623 10.1021/acs.chemrev.6b00346

[3] A. H. Chughtai , N. Ahmad , H. A. Younus , A. Laypkov , F. Verpoort , Chem. Soc. Rev. 2015, 44, 6804. 25958955 10.1039/c4cs00395k

[4] P. Horcajada , T. Chalati , C. Serre , B. Gillet , C. Sebrie , T. Baati , J. F. Eubank , D. Heurtaux , P. Clayette , C. Kreuz , J.‐S. Chang , Y. K. Hwang , V. Marsaud , P.‐N. Bories , L. Cynober , S. Gil , G. Ferey , P. Couvreur , R. Gref , Nat. Mater. 2010, 9, 172. 20010827 10.1038/nmat2608

[5] Y.‐B. Huang , J. Liang , X.‐S. Wang , R. Cao , Chem. Soc. Rev. 2017, 46, 126. 27841411 10.1039/c6cs00250a

[6] S. Qiu , M. Xue , G. Zhu , Chem. Soc. Rev. 2014, 43, 6116. 24967810 10.1039/c4cs00159a

[7] P. Silva , S. M. F. Vilela , J. P. C. Tome , F. A. Almeida Paz , Chem. Soc. Rev. 2015, 44, 6774. 26161830 10.1039/c5cs00307e

[8] X.‐P. Wu , L. Gagliardi , D. G. Truhlar , J. Chem. Phys. 2018, 150, 041701. 10.1063/1.5043538 30709313

[9] M. D. Allendorf , C. A. Bauer , R. K. Bhakta , R. J. T. Houk , Chem. Soc. Rev. 2009, 38, 1330. 19384441 10.1039/b802352m

[10] Y. Cui , Y. Yue , G. Qian , B. Chen , Chem. Rev. 2012, 112, 1126. 21688849 10.1021/cr200101d

[11] M. Gutiérrez , R. Navarro , F. Sánchez , A. Douhal , Phys. Chem. Chem. Phys. 2017, 19, 16337. 28569307 10.1039/c7cp02590d

[12] Z. Hu , B. J. Deibert , J. Li , Chem. Soc. Rev. 2014, 43, 5815. 24577142 10.1039/c4cs00010b

[13] L. E. Kreno , K. Leong , O. K. Farha , M. Allendorf , R. P. Van Duyne , J. T. Hupp , Chem. Rev. 2012, 112, 1105. 22070233 10.1021/cr200324t

[14] R. Li , Y.‐P. Yuan , L.‐G. Qiu , W. Zhang , J.‐F. Zhu , Small 2012, 8, 225. 22114057 10.1002/smll.201101699

[15] W. P. Lustig , S. Mukherjee , N. D. Rudd , A. V. Desai , J. Li , S. K. Ghosh , Chem. Soc. Rev. 2017, 46, 3242. 28462954 10.1039/c6cs00930a

[16] I. Stassen , N. Burtch , A. Talin , P. Falcaro , M. Allendorf , R. Ameloot , Chem. Soc. Rev. 2017, 46, 3185. 28452388 10.1039/c7cs00122c

[17] B. Wang , X.‐L. Lv , D. Feng , L.‐H. Xie , J. Zhang , M. Li , Y. Xie , J.‐R. Li , H.‐C. Zhou , J. Am. Chem. Soc. 2016, 138, 6204. 27090616 10.1021/jacs.6b01663

[18] M. Gutierrez , B. Cohen , F. Sánchez , A. Douhal , Phys. Chem. Chem. Phys. 2016, 18, 27761. 27731427 10.1039/c6cp03791g

[19] M. Gutiérrez , F. Sánchez , A. Douhal , J. Mater. Chem. C 2015, 3, 11300.

[20] W. A. Maza , R. Padilla , A. J. Morris , J. Am. Chem. Soc. 2015, 137, 8161. 26043760 10.1021/jacs.5b03071

[21] A. Van Wyk , T. Smith , J. Park , P. Deria , J. Am. Chem. Soc. 2018, 140, 2756. 29421871 10.1021/jacs.7b13211

[22] K. Hendrickx , D. E. P. Vanpoucke , K. Leus , K. Lejaeghere , A. Van Yperen‐De Deyne , V. Van Speybroeck , P. Van Der Voort , K. Hemelsoet , Inorg. Chem. 2015, 54, 10701. 26540517 10.1021/acs.inorgchem.5b01593

[23] Z. Hu , D. Zhao , Dalton Trans. 2015, 44, 19018. 26478120 10.1039/c5dt03359d

[24] R. J. Marshall , R. S. Forgan , Eur. J. Inorg. Chem. 2016, 2016, 4310.

[25] A. S. Yasin , J. Li , N. Wu , T. Musho , Phys. Chem. Chem. Phys. 2016, 18, 12748. 27098230 10.1039/c5cp08070c

[26] P. Deria , J. Yu , T. Smith , R. P. Balaraman , J. Am. Chem. Soc. 2017, 139, 5973. 28385020 10.1021/jacs.7b02188

[27] W.‐G. Liu , D. G. Truhlar , Chem. Mater. 2017, 29, 8073.

[28] J. Yu , X. Li , P. Deria , ACS Sustainable Chem. Eng. 2019, 7, 1841.

[29] J. Yu , J. Park , A. Van Wyk , G. Rumbles , P. Deria , J. Am. Chem. Soc. 2018, 140, 10488. 30040404 10.1021/jacs.8b04980

[30] X.‐P. Wu , L. Gagliardi , D. G. Truhlar , J. Am. Chem. Soc. 2018, 140, 7904. 29807431 10.1021/jacs.8b03613

[31] S. Smolders , A. Struyf , H. Reinsch , B. Bueken , T. Rhauderwiek , L. Mintrop , P. Kurz , N. Stock , D. E. De Vos , Chem. Commun. 2018, 54, 876. 10.1039/c7cc08200b 29236104

[32] K. M. Choi , D. Kim , B. Rungtaweevoranit , C. A. Trickett , J. T. D. Barmanbek , A. S. Alshammari , P. Yang , O. M. Yaghi , J. Am. Chem. Soc. 2017, 139, 356. 28004911 10.1021/jacs.6b11027

[33] T. H. El‐Assaad , M. Auer , R. Castañeda , K. M. Hallal , F. M. Jradi , L. Mosca , R. S. Khnayzer , D. Patra , T. V. Timofeeva , J.‐L. Brédas , E. J. W. List‐Kratochvil , B. Wex , B. R. Kaafarani , J. Mater. Chem. C 2016, 4, 3041.

[34] L. E. Brady , Handbook of Fluorescence Spectra of Aromatic Molecules, Academic Press, New York 1971.

[35] M. Gutiérrez , F. Sánchez , A. Douhal , Chem. ‐ Eur. J. 2016, 22, 13072. 27404091 10.1002/chem.201600669

[36] J. B. Birks , D. J. Dyson , I. H. Munro , H. Flowers Brian , Proc. R. Soci. London, Ser. A 1963, 275, 575.

[37] M. Gutiérrez , C. Martin , K. Kennes , J. Hofkens , M. Van der Auweraer , F. Sanchez , A. Douhal , Adv. Opt. Mater. 2018, 6, 1701060.

[38] L. Liu , Z. A.‐O. Chen , J. A.‐O. X. Wang , D. A.‐O. Zhang , Y. Zhu , S. A.‐O. Ling , K. A.‐O. Huang , Y. A.‐O. Belmabkhout , K. A.‐O. Adil , Y. Zhang , B. A.‐O. Slater , M. A.‐O. Eddaoudi , Y. A.‐O. Han , Nat. Chem. 2019, 11, 622. 31086300 10.1038/s41557-019-0263-4

[39] Z. Fang , B. Bueken , D. E. De Vos , R. A. Fischer , Angew. Chem., Int. Ed. 2015, 54, 7234. 10.1002/anie.201411540 PMC4510710 26036179

[40] M. Lammert , Mt. Wharmby , S. Smolders , B. Bueken , A. Lieb , K. A. Lomachenko , D. D. Vos , N. Stock , Chem. Commun. 2015, 51, 12578. 10.1039/c5cc02606g 26154160

[41] R. D'Amato , A. Donnadio , M. Carta , C. Sangregorio , D. Tiana , R. Vivani , M. Taddei , F. Costantino , ACS Sustainable Chem. Eng. 2019, 7, 394.

[42] N. Heidenreich , S. Waitschat , H. Reinsch , Z. Anorg. Allg. Chem. 2018, 644, 1826.

[43] S. Smolders , K. A. Lomachenko , B. Bueken , A. Struyf , A. L. Bugaev , C. Atzori , N. Stock , C. Lamberti , M. B. J. Roeffaers , D. A.‐O. De Vos , ChemPhysChem 2018, 19, 373. 29027736 10.1002/cphc.201700967

[44] M. de Miguel , F. Ragon , T. Devic , C. Serre , P. Horcajada , H. Garcia , ChemPhysChem 2012, 13, 3651. 22907833 10.1002/cphc.201200411

[45] C. Gomes Silva , I. Luz , F. X. Llabrés i Xamena , A. Corma , H. García , Chem. ‐ Eur. J. 2010, 16, 11133. 20687143 10.1002/chem.200903526

[46] K. G. M. Laurier , E. Fron , P. Atienzar , K. Kennes , H. Garcia , M. Van der Auweraer , D. E. De Vos , J. Hofkens , M. B. J. Roeffaers , Phys. Chem. Chem. Phys. 2014, 16, 5044. 24481514 10.1039/c3cp55028a

[47] T. Tachikawa , J. R. Choi , M. Fujitsuka , T. Majima , J. Phys. Chem. C 2008, 112, 14090.

[48] G. Cao , C. J. Brinker , Annual review of nano research, Vol. 2, World Scientific, Hackensack, NJ 2008.

[49] A. Dhakshinamoorthy , A. M. Asiri , H. García , Angew. Chem., Int. Ed. 2016, 55, 5414. 10.1002/anie.201505581 26970539

[50] L. Hanna , P. Kucheryavy , C. Liu , X. Zhang , J. V. Lockard , J. Phys. Chem. C 2017, 121, 13570.

[51] T. Zhang , Y. Jin , Y. Shi , M. Li , J. Li , C. Duan , Coord. Chem. Rev. 2019, 380, 201.

[52] J. R. Choi , T. Tachikawa , M. Fujitsuka , T. Majima , Langmuir 2010, 26, 10437. 20515053 10.1021/la101770h

[53] L. Hu , X.‐J. Hong , X.‐M. Lin , J. Lin , Q.‐X. Cheng , B. Lokesh , Y.‐P. Cai , Cryst. Growth Des. 2018, 18, 7088.

[54] A. Lan , K. Li , H. Wu , D. H. Olson , T. J. Emge , W. Ki , M. Hong , J. Li , Angew. Chem., Int. Ed. 2009, 48, 2334. 10.1002/anie.200804853 19180622

[55] S. S. Nagarkar , B. Joarder , A. K. Chaudhari , S. Mukherjee , S. K. Ghosh , Angew. Chem., Int. Ed. 2013, 52, 2881. 10.1002/anie.201208885 23355359

[56] J. R. Lakowicz , Principles of Fluorescence Spectroscopy, 2nd ed., Kluwer Academic/Plenum, New York 1999.

[57] S. L. Estes , M. R. Antonio , L. Soderholm , J. Phys. Chem. C 2016, 120, 5810.

[58] J. A. Organero , L. Tormo , A. Douhal , Chem. Phys. Lett. 2002, 363, 409.

[59] C. Randino , M. Ziółek , R. Gelabert , J. A. Organero , M. Gil , M. Moreno , J. M. Lluch , A. Douhal , Phys. Chem. Chem. Phys. 2011, 13, 14960. 21755097 10.1039/c1cp21039d

[60] M. Gil , A. Douhal , Chem. Phys. 2008, 350, 179.

[61] N. Alarcos , B. Cohen , A. Douhal , J. Phys. Chem. C 2014, 118, 19431. 10.1021/jp505591k 25060093

