# Supplementary material for: Unravelling Why and to What Extent the Topology of Similar Ce‐Based MOFs Conditions their Photodynamic: Relevance to Photocatalysis and Photonics
Source: Adv Sci (Weinh). 2019 Jul 31;6(19):1901020. doi: 10.1002/advs.201901020 (PMC6774026; doi:10.1002/advs.201901020)
Supplement: Supplementary file 1 — Supplementary [file ADVS-6-1901020-s001.pdf]

Copyright WILEY-VCH Verlag GmbH & Co. KGaA, 69469 Weinheim, Germany, 2019.

Supporting Information

for *Adv. Sci.,* DOI: 10.1002/advs.201901020

Unravelling Why and to What Extent the Topology of Similar Ce-Based MOFs Conditions their Photodynamic: Relevance to Photocatalysis and Photonics

*Elena Caballero-Mancebo, Boiko Cohen, Simon Smolders, Dirk E.De Vos, and Abderrazzak Douhal**

Supporting Information

for

Unravelling Why and to What Extent the Topology of Similar Ce-based MOFs Conditions their Photodynamic: Relevance to Photocatalysis and Photonics

Elena Caballero-Mancebo[a], Boiko Cohen[a], Simon Smolders[b], Dirk E. De Vos[b] and Abderrazzak Douhal[a*]

[a]Departamento de Química Física, Facultad de Ciencias Ambientales y Bioquímica, and INAMOL, Universidad de Castilla-La Mancha, Avenida Carlos III, S/N, 45071 Toledo, Spain.

[b] Centre for Membrane Separations, Adsorption, Catalysis and Spectroscopy for Sustainable Solutions, Department M²S, KU Leuven, Celestijnenlaan 200F p.o. box 2461, 3001 Leuven, Belgium.

*corresponding author: Abderrazzak.Douhal@uclm.es

Figures S1-S22 in pages 5-16, and Table S1-S5 in pages 17 to 20.

Spectroscopic and Dynamic Measurements

The picosecond time-resolved emission experiments have been performed employing a time-correlated single-photon counting (TSCPC) system.[1] The samples were excited by a 40 ps-pulsed (~1 mW, 40 MHz repetition rate) diode-laser (PicoQuant) centered at 370 nm. The measured instrument response function (IRF) of the set-up is ~70 ps. The fluorescence signal was collected at the magic angle (54.74°) and monitored at a 90° angle to the excitation beam at discrete emission wavelengths. The decays were deconvoluted and fitted to a multiexponential function using the FLUOFIT package (PicoQuant) allowing single and global fits. The quality of the fits as well as the number of exponentials were carefully selected based on the reduced χ^2 values (which were always below <1.2) and the distributions of the residuals.

The confocal microscopy measurements were performed on a MicroTime 200 confocal microscope (PicoQuant). The excitation was conducted with the same diode laser used in the TSCPC experiment. The emission signal was collected using a 430-nm long pass filter (Chroma). In brief, the instrument consists of an inverse Olympus IX 71 microscope equipped with a water-immersion objective (x60 NA1.2, Olympus) and a 2D piezo scanner (Physik Instrumente). The emitted light was then focused on a 50-μm pinhole and later collimated to two independent single-photon avalanche photodiodes (Micro-Photon-Devices) for time-resolved measurements. The emission spectra were collected through a Shamrock ST-303i (Andor Technology) imaging spectrograph and detected by an Andor Newton EMCCD camera (Andor Technology). The samples were measured in the solid state, which was prepared by spilling a small amount of the solid powder over the coverslip and introducing it into the sample holder. All the experiments were done at room temperature (295 K).

The nanosecond (ns) flash photolysis setup has been described previously.[2] Briefly, it consists of a LKS.60 laser flash photolysis spectrometer (Applied Photophysics) and a Vibrant (HE) 355 II laser (Opotek). To excite the sample, we used the signal from an OPO (pumped by a Q-switched Nd:YAG laser, Brilliant, Quantel) at 430 nm. The pump fluence was 80 mJ cm^2. As a probe source, we used the output of a 150 W Xenon arc lamp. The light transmitted through the sample was then dispersed by a monochromator and detected by a visible photomultiplier (Applied Photophysics R928), coupled to a digital oscilloscope (Agilent Infiniium DS08064A, 600 MHz, 4 GSa s1). The measured IRF of the system was ~8 ns. All the experiments were performed at 293 K.

Femtosecond time-resolved emission transients were collected using the fluorescence up-conversion technique.[3] The system consists of a femtosecond Ti:Sapphire MaiTai HP (Spectra Physics) laser that generates the excitation beam at 370 nm (~5-10 mW). The polarization of the latter was set to magic angle with respect to the fundamental beam. The sample has been placed in a 1 mm thick rotating cell. The fluorescence was focused with reflective optics into a 0.5 mm BBO crystal and gated with the fundamental femtosecond beam. The IRF of the apparatus (measured as a Raman signal of pure solvent) was ~270 fs (full width at half-maximum, FWHM) at the excitation wavelength. To analyze the decays, a multi-exponential function convoluted with the IRF was used to fit the experimental data. In all cases, the errors for the calculated time components were smaller than 15%.

The fs-transient UV-visible-NIR absorption experiments were done using a chirped pulse amplification setup.[4] In brief, it comprises a Ti:Sapphire oscillator (TISSA 50, CDP Systems) pumped by a 5 W diode laser (Verdi 5, Coherent) that seeds (800 nm, 30 fs, 450 mW at 86 MHz) a regenerative amplifier (Legend-USP, Coherent).

The amplified fundamental beam (50 fs, 1 W at 1 kHz) pumps an optical parametric amplifier for wavelength conversion (CDP Systems). The second harmonic (400 nm) of the fundamental beam or the fourth harmonic (350 nm) of the OPA output was used as the pump. The pump pulse intensity was kept below ~200 mW. The instrument response function (IRF) was measured in terms of ΔOD for the Raman scattering of acetone following excitation at 400 nm and to give 120 fs, respectively. The samples were placed in a 1 mm spinning cell to avoid photodegradation.

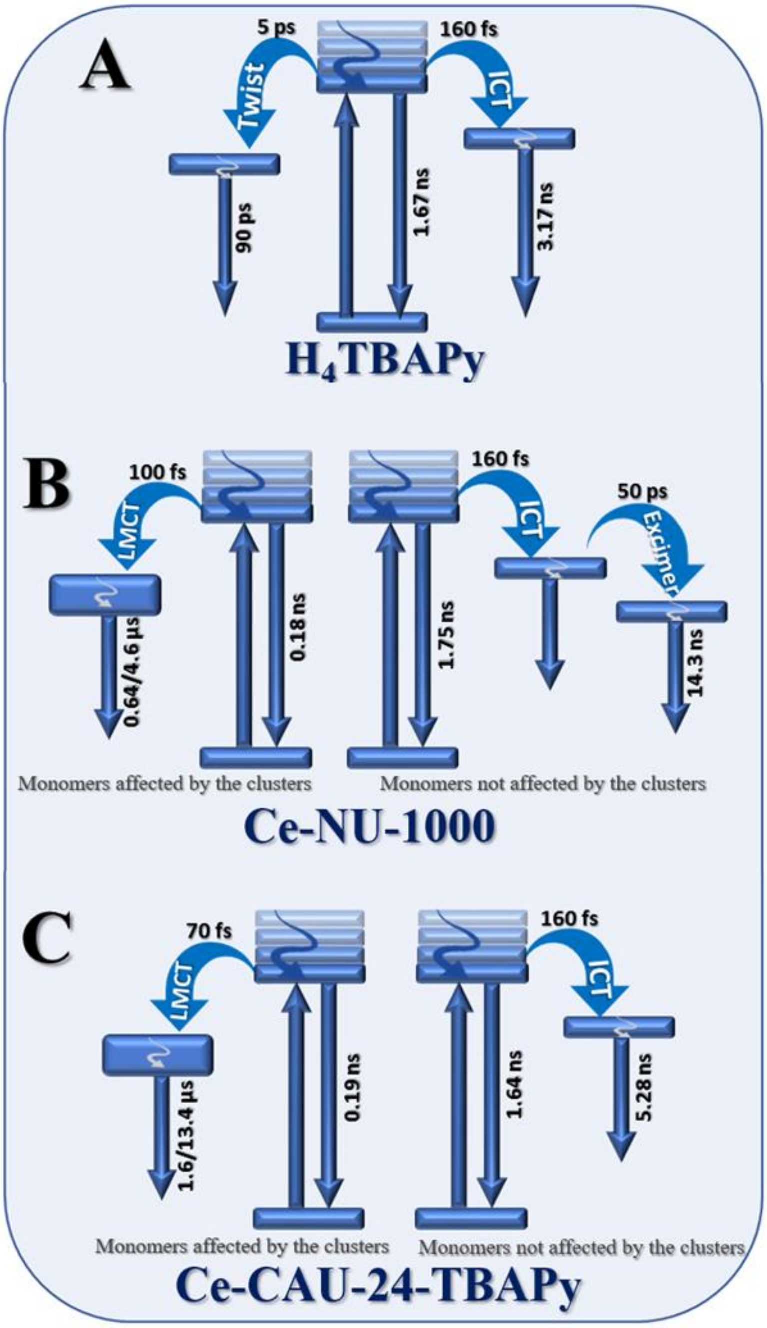

Scheme S1. Representation (not in scale) of the photodynamical behavior of (A) H_4TBAPy, (B) Ce-NU-1000 and (C) Ce-CAU-24-TBAPy in acetone.

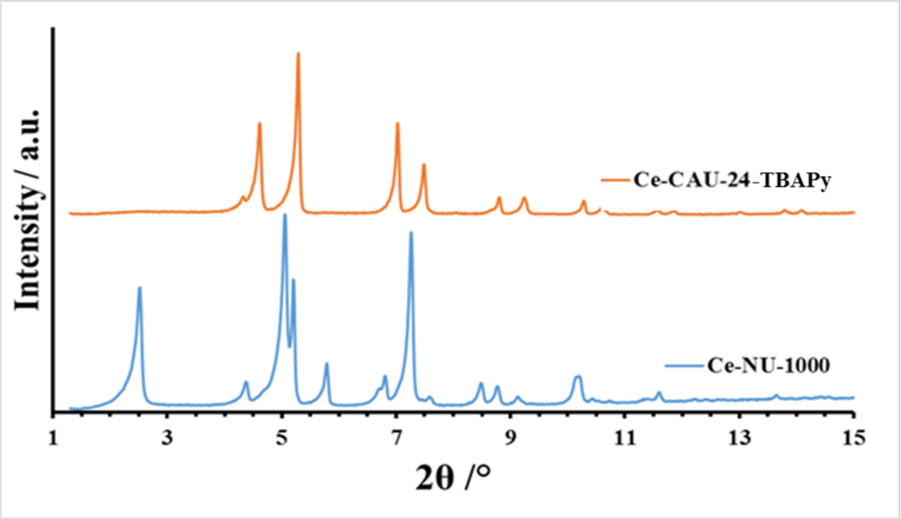

Figure S1. X-Ray diffraction patterns of Ce-CAU-24-TBAPy and Ce-NU-1000.

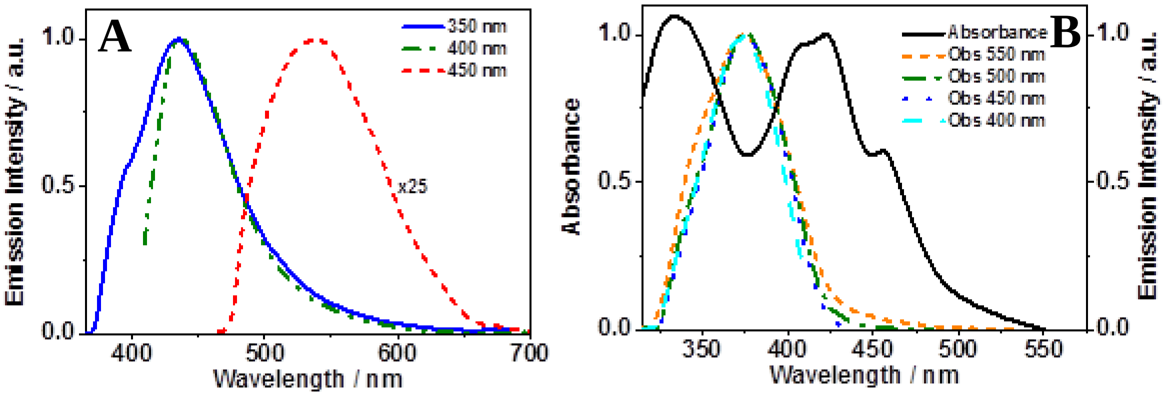

Figure S2. (A) Emission spectra of H_4TBAPy in acetone at different excitation wavelengths. (B) Comparison of UV-visible absorption and excitation spectra gated at different observation wavelengths.

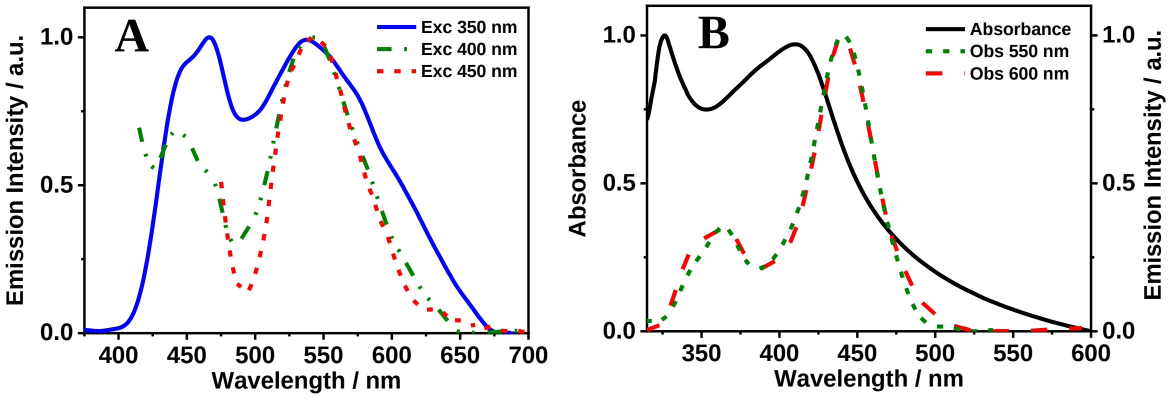

Figure S3. (A) Emission spectra of Ce-NU-1000 in acetone at different excitation wavelengths. (B) Comparison of the UV-visible absorption spectrum with the excitation spectra gated at

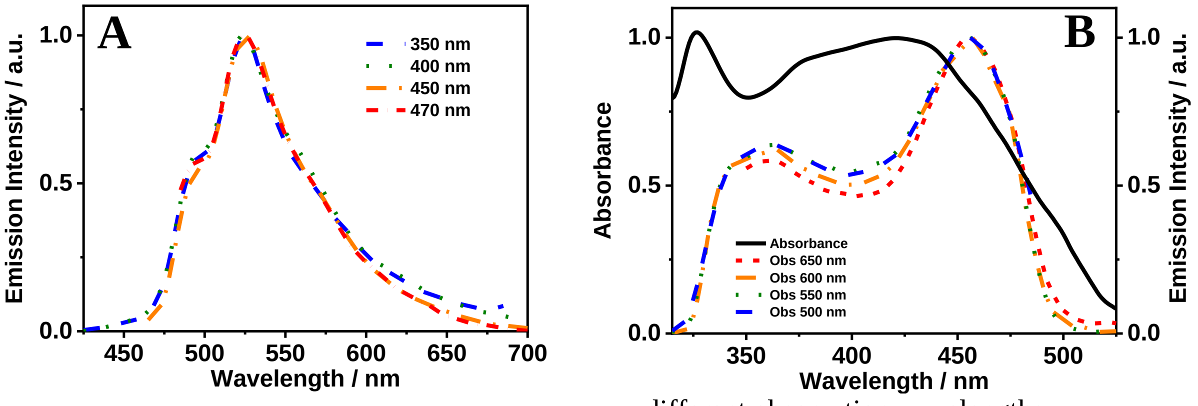

different observation wavelengths.

Figure S4. (A) Emission spectra of Ce-CAU-24-TBAPy in acetone at different excitation wavelengths. (B) Comparison of the UV-visible absorption spectrum with the excitation spectra gated at different observation wavelengths.

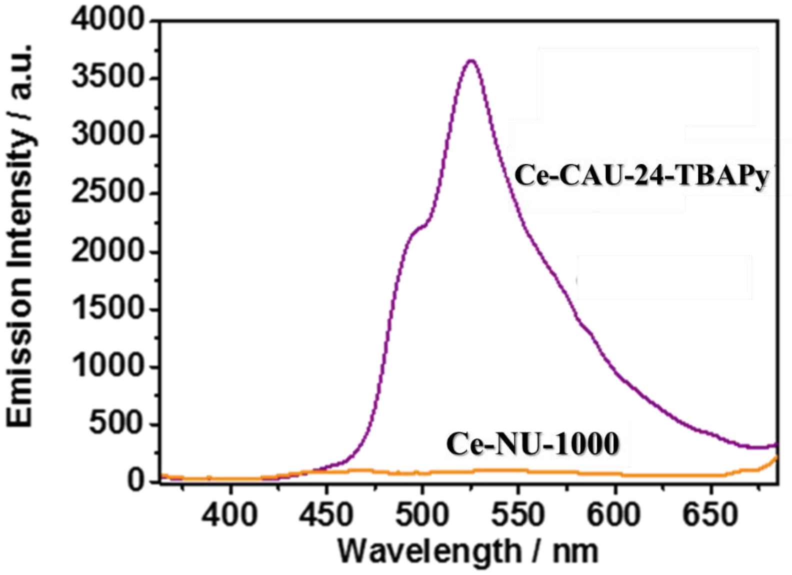

Figure S5. Not normalized visible emission spectra of Ce-CAU-24-TBAPy (purple line) and Ce-NU-1000 (orange line) in acetone suspension, upon excitation at 350 nm. The samples have the same optical density at 425 nm.

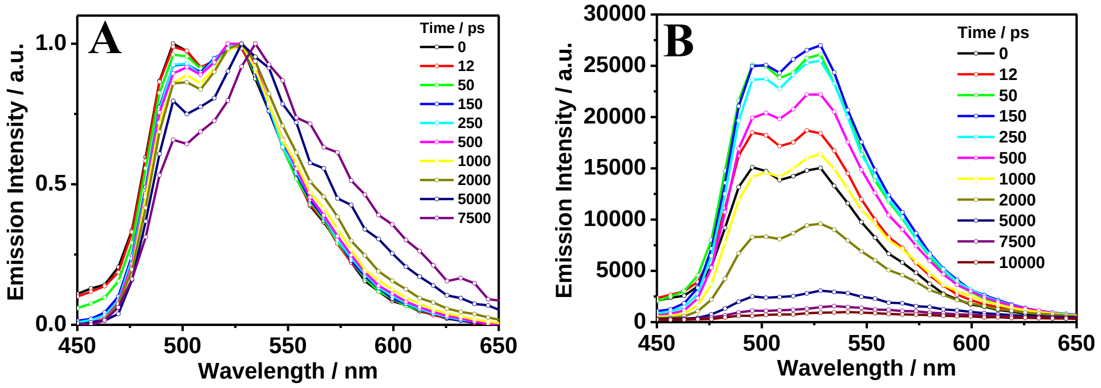

Figure S6. (A) Normalized and (B) not normalized ps-time-resolved emission spectra of Ce-CAU-24-TBAPy in acetone upon excitation at 370 nm and observation at different gating times as indicated in the insets.

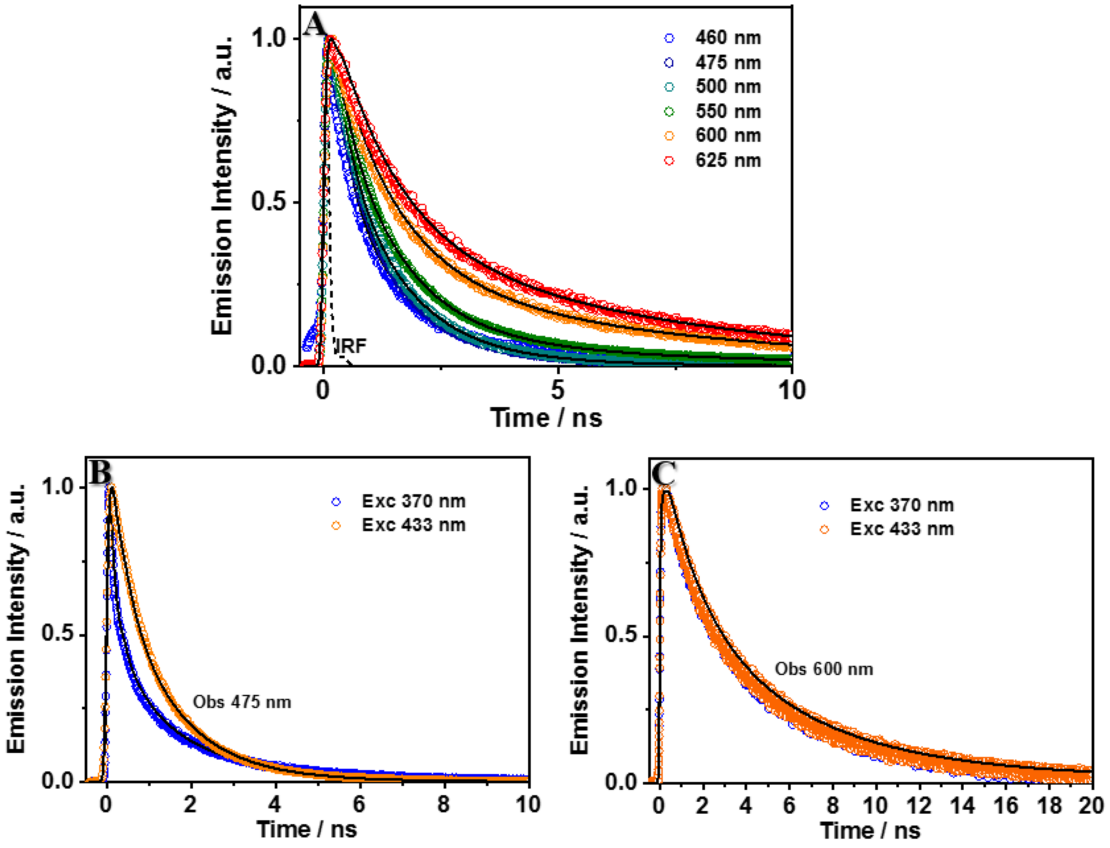

Figure S7. (A) Magic-angle emission decays of Ce-CAU-24-TBAPy in acetone upon excitation at 430 nm and observation at the indicated wavelengths. (B) and (C) comparison of the emission decays upon excitation at 370 and 433 nm and observation at 475 and 600 nm, respectively.

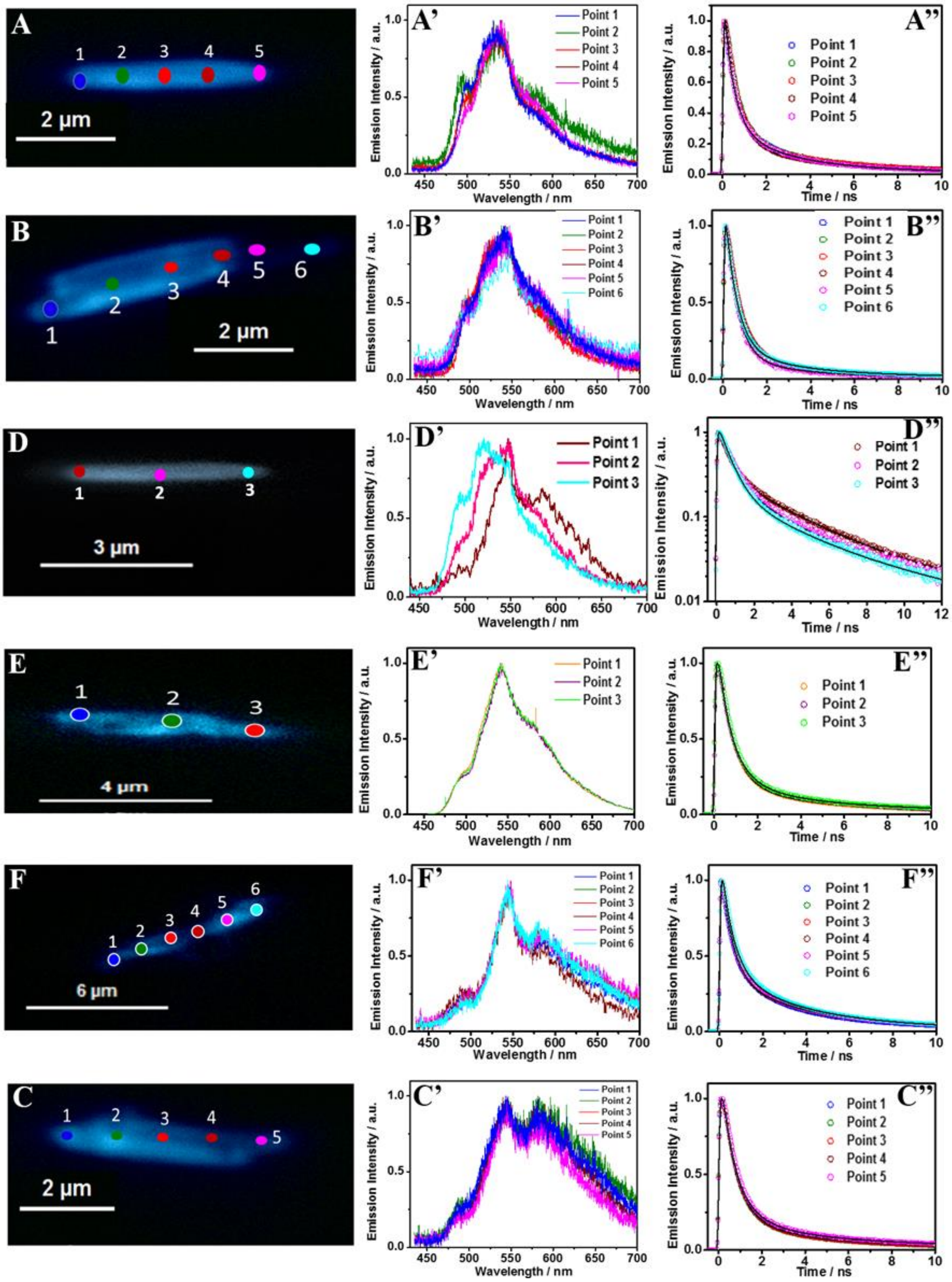

Figure S8. Fluorescence spectra and decays of different Ce-CAU-24-TBAPy single crystals observed at the selected points as indicated.

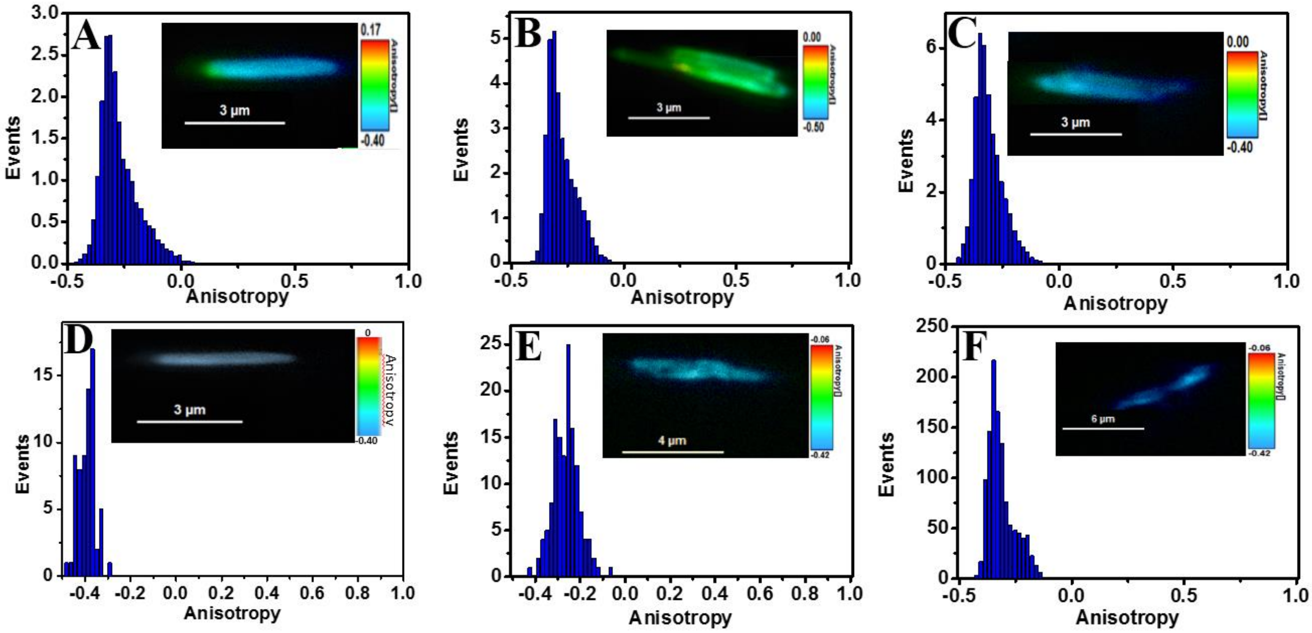

Figure S9. Histograms of the emission anisotropy of different single crystals of Ce-CAU-24-TBAPy. The inset shows an image of the studied crystal.

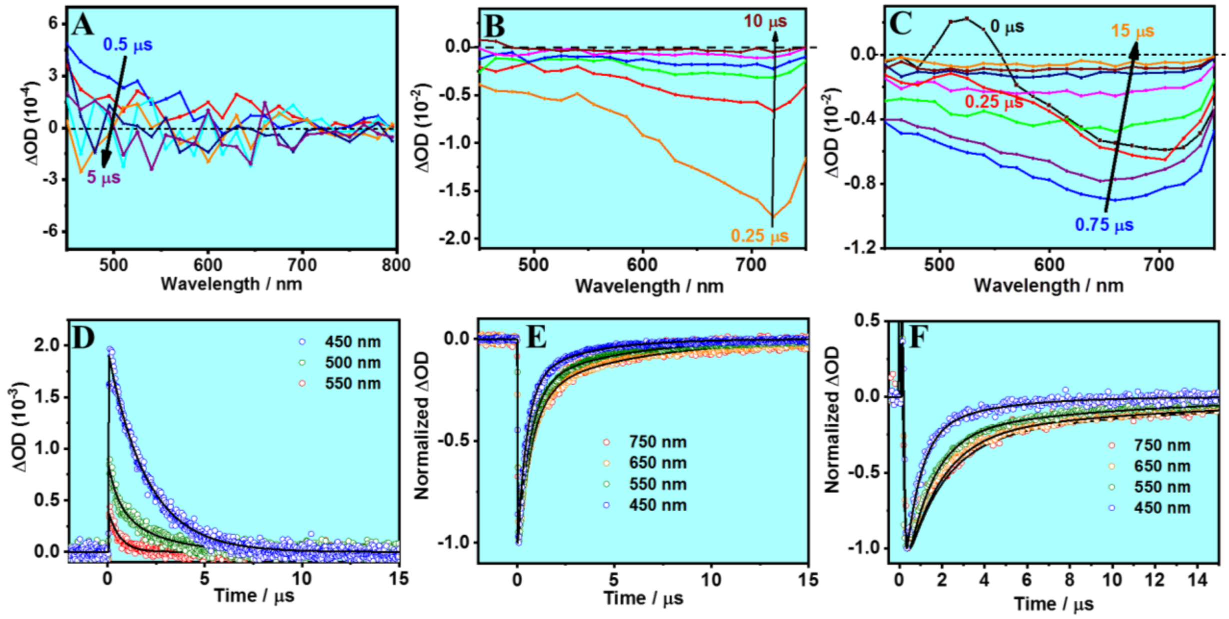

Figure S10. (A-C) μs transient absorption spectra and (D-F) decays gated at different times and probing at different wavelengths, respectively. (A and D) for the linker, (B and E) for Ce-NU-1000 and (C and F) for Ce-CAU-24-TBAPy. The excitation wavelength was 430 nm.

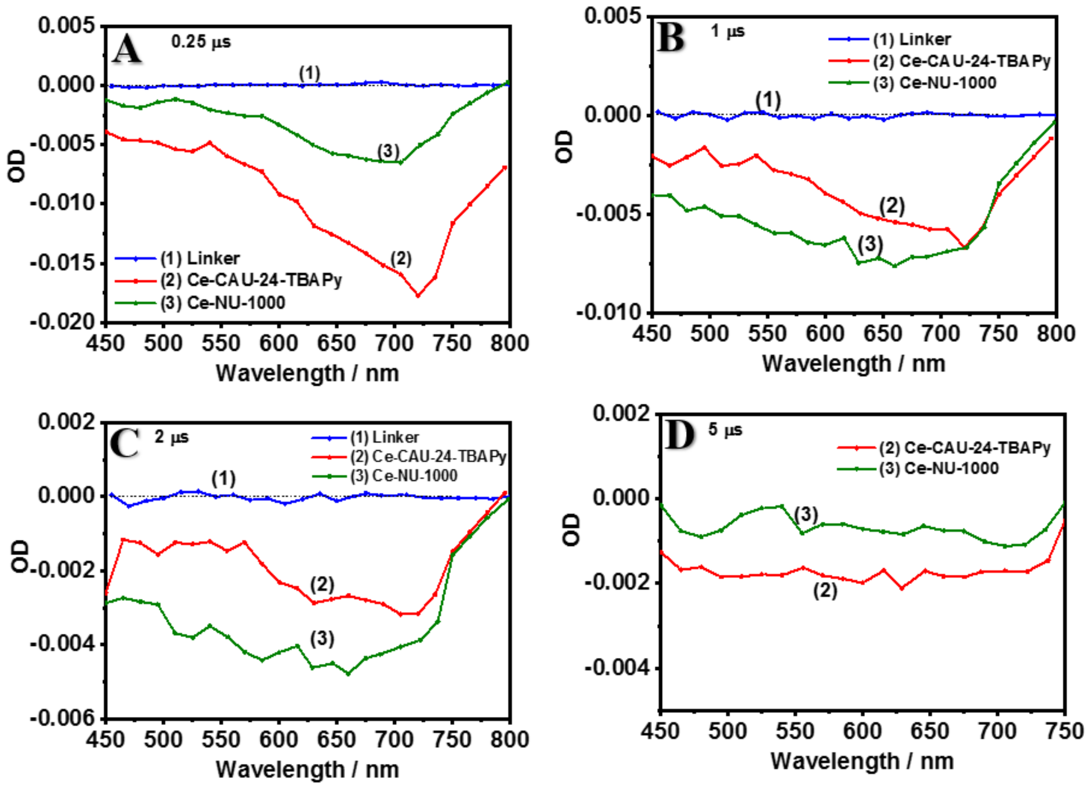

Figure S11. Time-resolved transient absorption spectra of the free linker (blue lines, 1), Ce-CAU-24-TBAPy (red lines, 2) and Ce-NU-1000 (green lines, 3) in acetone collected at different gating times: (A) 0.25 µs, (B) 1 µs, (C) 2 µs and (D) 5 µs upon excitation at 430 nm.

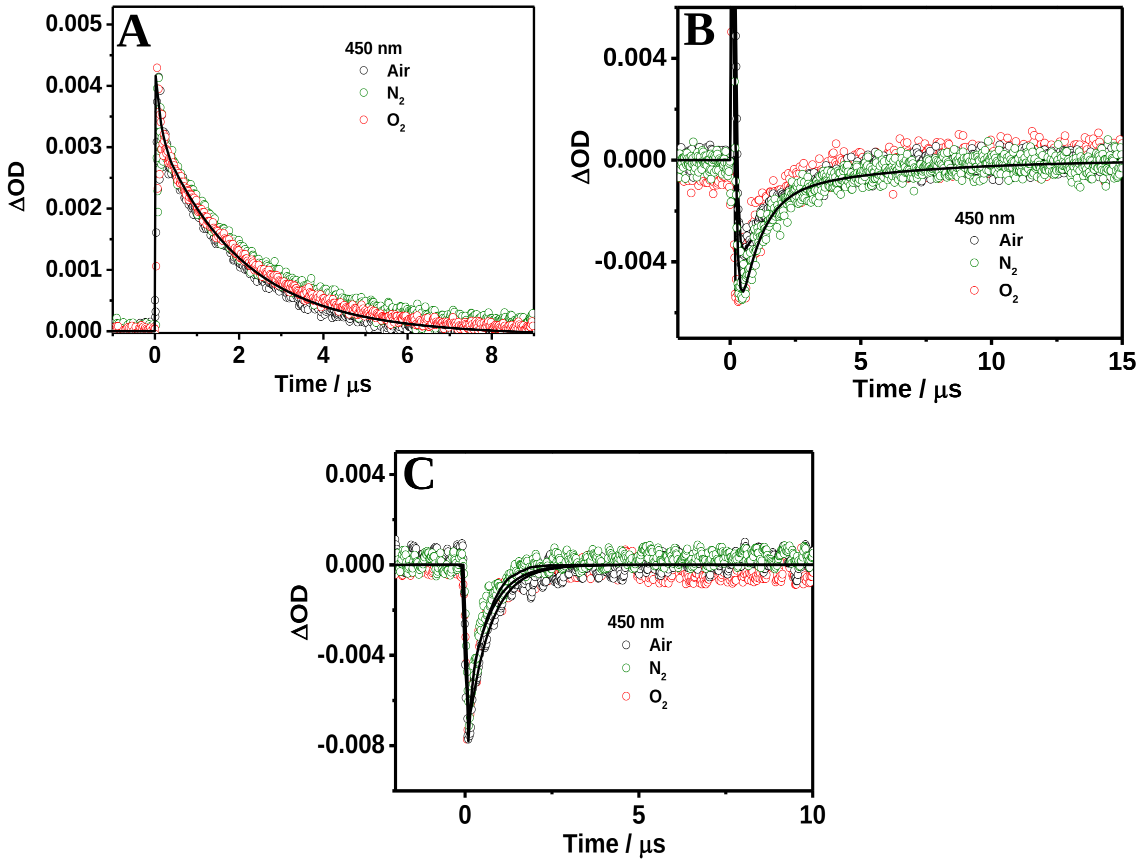

Figure S12. Ms-transient visible absorption decays of (A) the linker, (B) Ce-CAU-24-TBAPy and (C) Ce-NU-1000 in acetone recorded upon excitation at 430 nm and observation at 450 nm under air atmosphere (black circles), nitrogen atmosphere (green circles) and oxygen atmosphere (red circles). We observed no oxygen affect excluding the involving of triplet state in those decays.

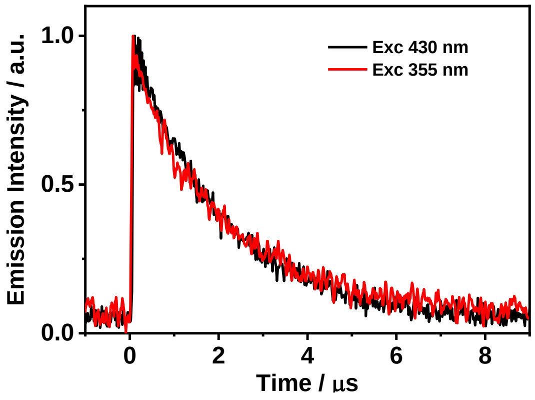

Figure S13. Comparison of normalized µs transients of the linker in acetone upon different excitation wavelengths and observation at 450 nm.

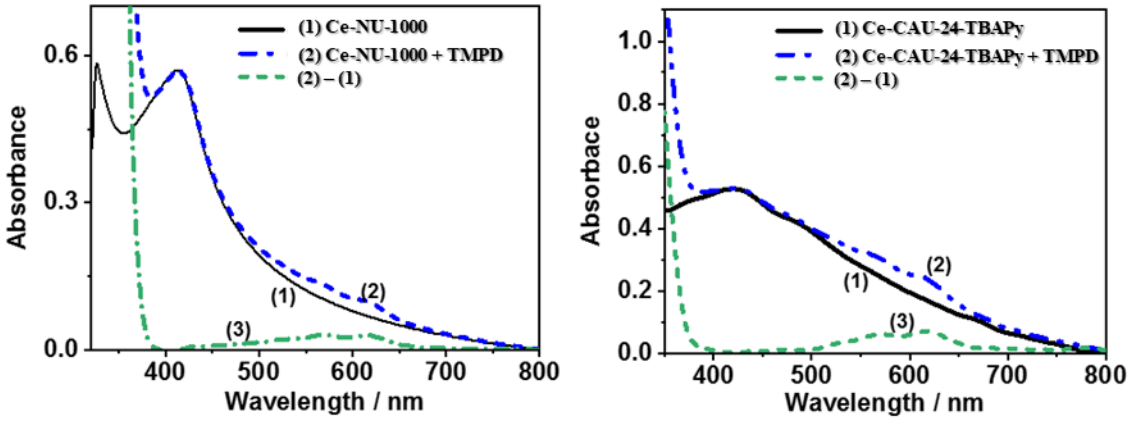

Figure S14. UV-visible absorption spectra of Ce-NU-1000 and Ce-CAU-24-TBAPy in acetone suspensions (1) without and (2) with the presence of TMPD, (3) is the result of the spectral difference: (2)-(1). The spectral change is instantaneous, with no need of irradiation.

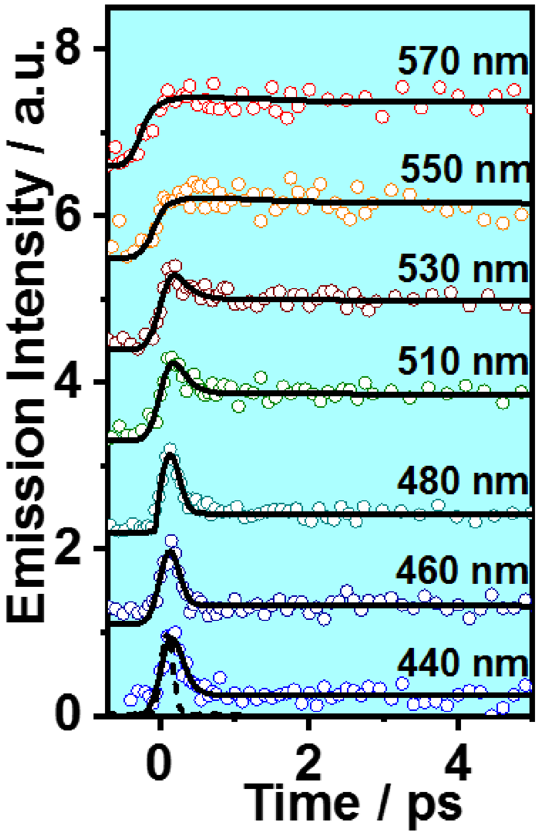

Figure S15. Femtosecond emission transients of Ce-CAU-24-TBAPy in acetone suspension upon excitation at 370 nm and observation at the indicated wavelengths. The solid black lines are for the best fit using multiexponential functions.

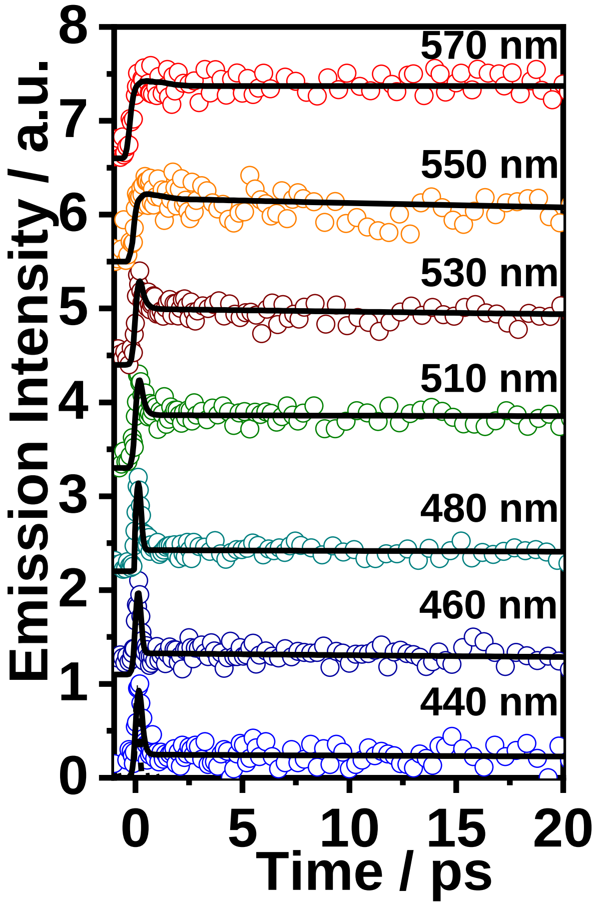

Figure S16. Femtosecond emission transients of Ce-CAU-24-TBAPy in an acetone suspension upon excitation at 370 nm and observation at the indicated wavelengths.

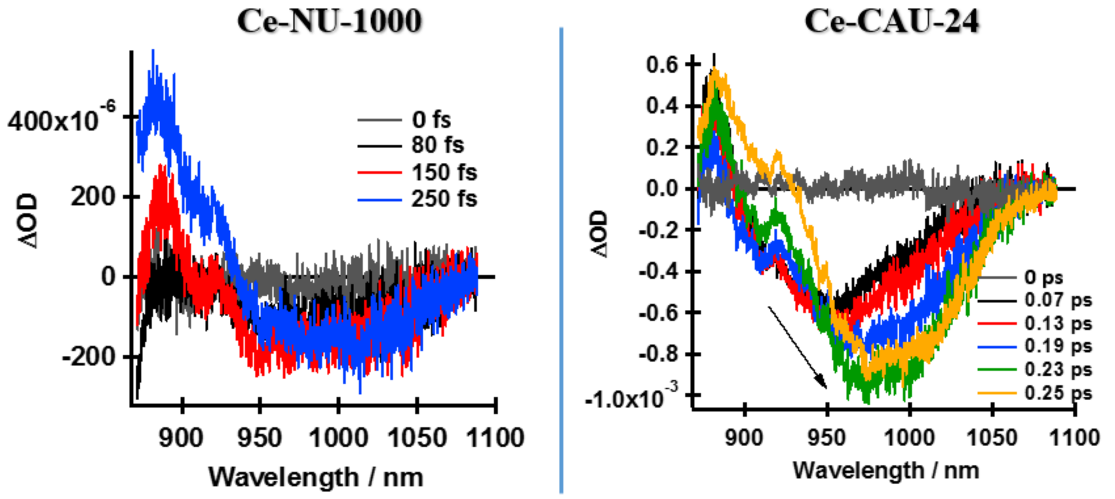

Figure S17. Time evolution of the fs-transients absorption spectra of Ce-NU-1000 and Ce-CAU-24-TBAPy in acetone suspensions in terms of the change in the optical density (ΔOD) upon excitation at 400 nm and recorded at the early pump-probe delay times.

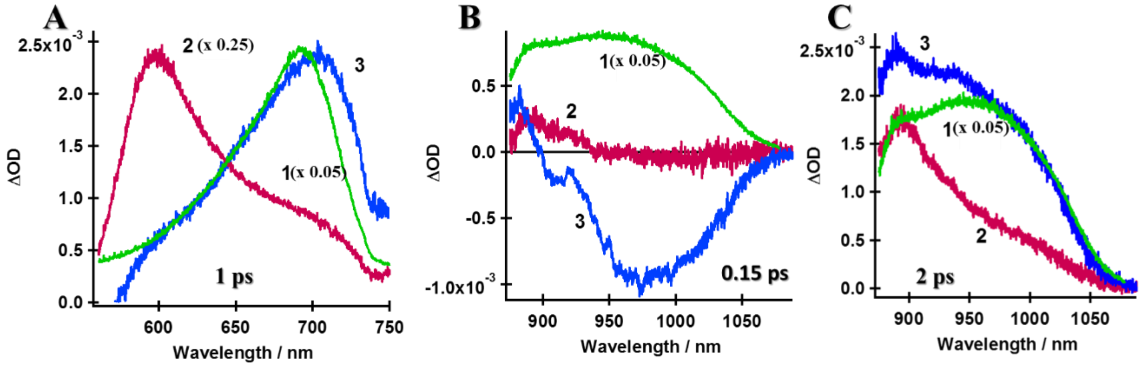

Figure S18. Time evolution of the fs-transients absorption spectra of (1) H_4TBAPy, (2) Ce-NU-1000 and (3) Ce-CAU-24-TBAPy in acetone at different gating times.

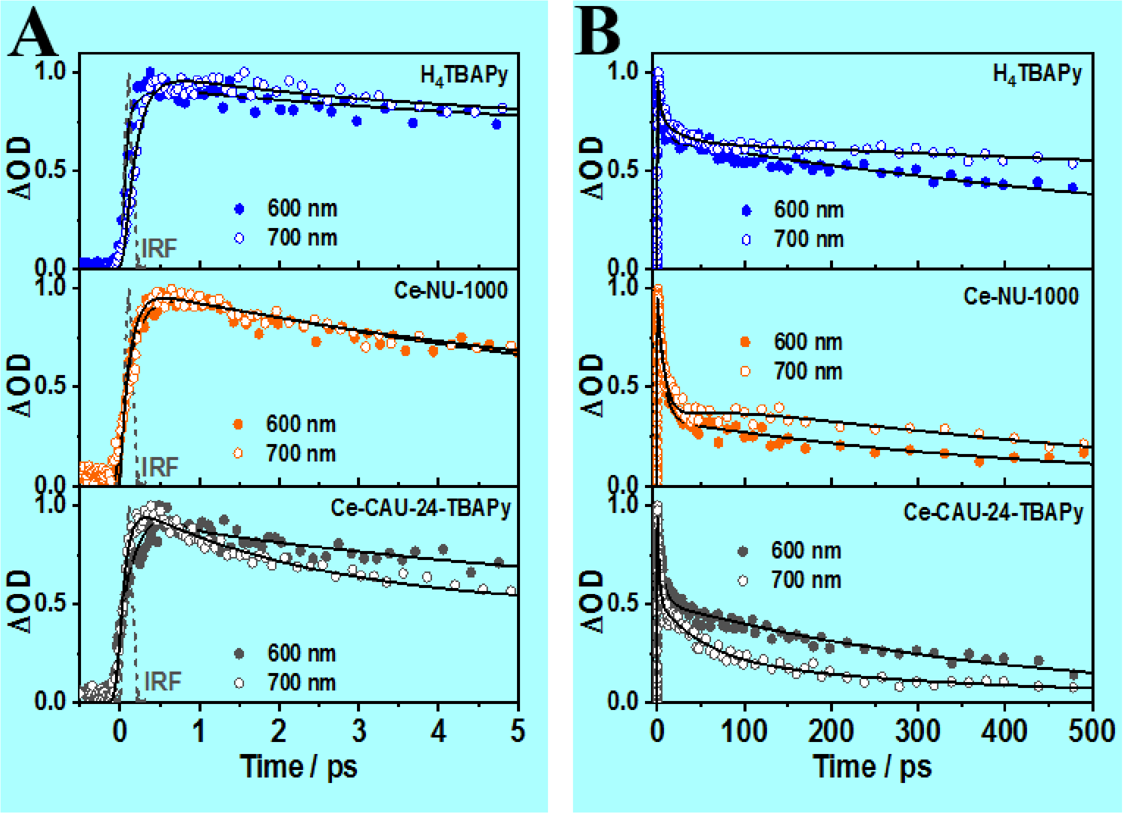

Figure S19. Femtosecond transient decays in terms of changes in the optical density (ΔOD) of Ce-CAU-24-TBAPy, Ce-NU-1000 in acetone suspensions and H_4TBAPy in (1:1) acetone:DMF solution. The excitation wavelength was 400 nm and the observation wavelengths are indicated in the inserts. The solid lines are from the best multiexponential fits (see Table 2 in the main text).

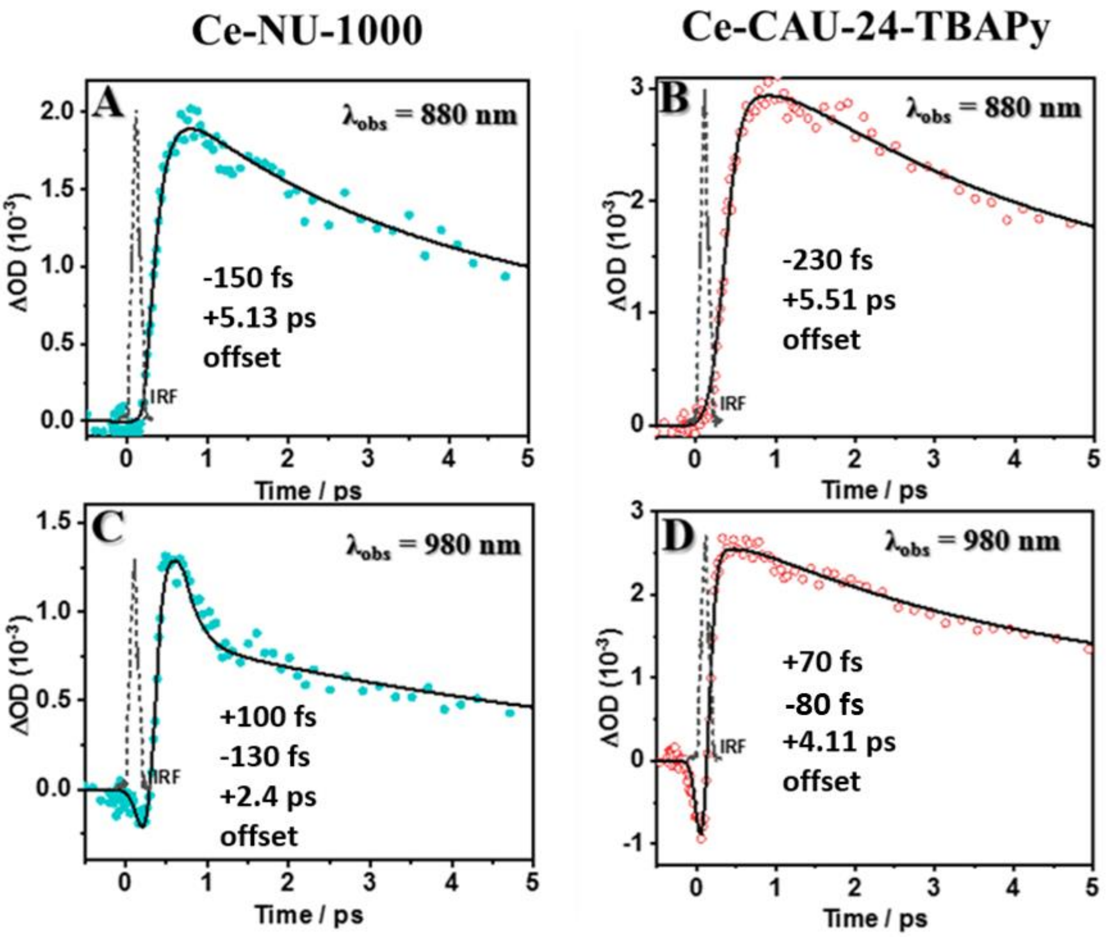

Figure S20. Femtosecond transient decays of Ce-NU-1000 and Ce-CAU-24-TBAPy in acetone suspensions upon excitation at 400 nm and gating at 880 and 980 nm. The solid lines are from the best multiexponential fits, which give the time constants inserted in the panels.

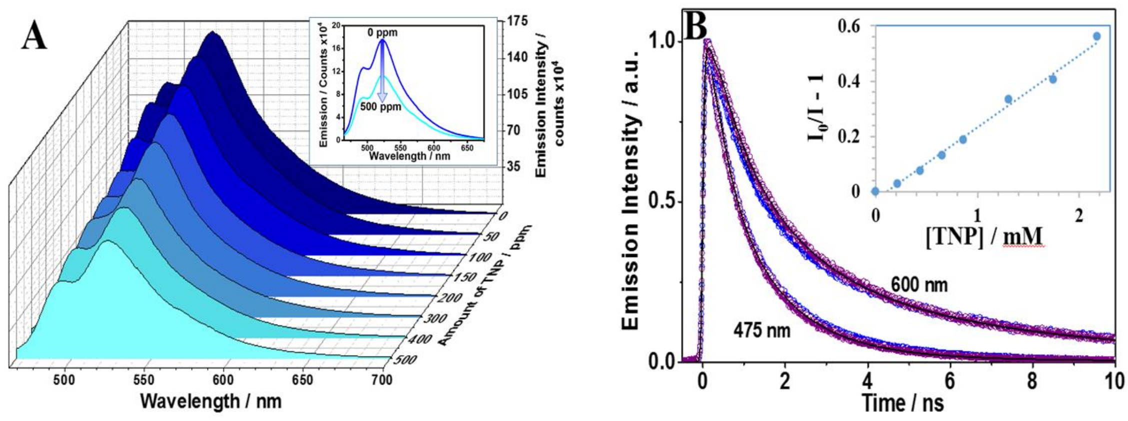

Figure S21. Emission spectra of Ce-CAU-24-TBAPy in acetone (3 mol) without and in presence of different amounts (0, 50, 100, 150, 200, 300, 400 and 500 ppm) of (A) trinitrophenol (TNP). The excitation wavelength was 450 nm. (B) Comparison of the emission decays of Ce-CAU-24-TBAPy in acetone suspension with and without 500 ppm of TNP, upon excitation at 370 m. The insert in (B) shows Stern-Volmer analysis of the emission quenching upon addition of TNP. $K_S = 2.26x10^2$ M^{-1}.

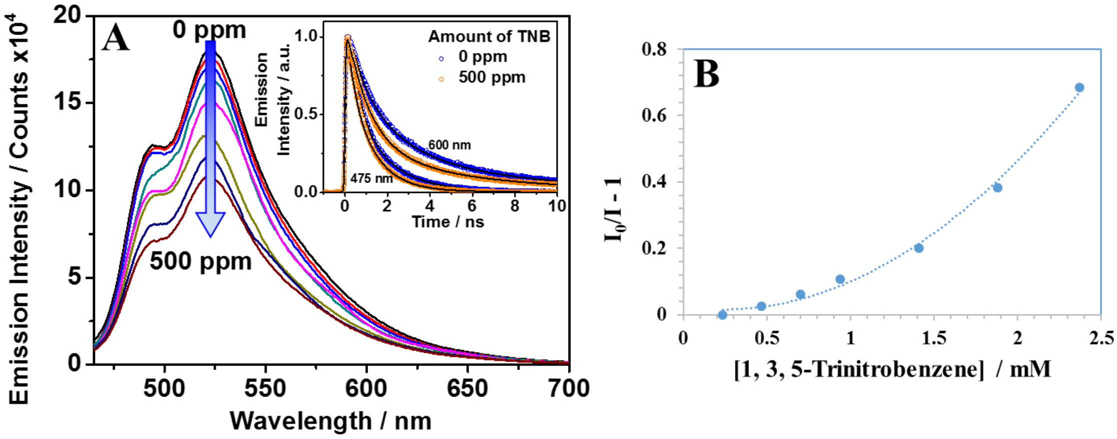

Figure S22. (A) Emission spectra of Ce-CAU-24-TBAPy in acetone without and in presence of different amounts (0, 50, 100, 150, 200, 300, 400 and 500 ppm) of 1, 3, 5-Trinitrotobencene (TNB). The excitation wavelength was 450 nm. The insert shows a comparison of the emission decays of the sample with and without containing 500 ppm of TNB, upon excitation at 370 m. (B) Stern-Volmer analysis of the emission quenching upon addition of TNB.

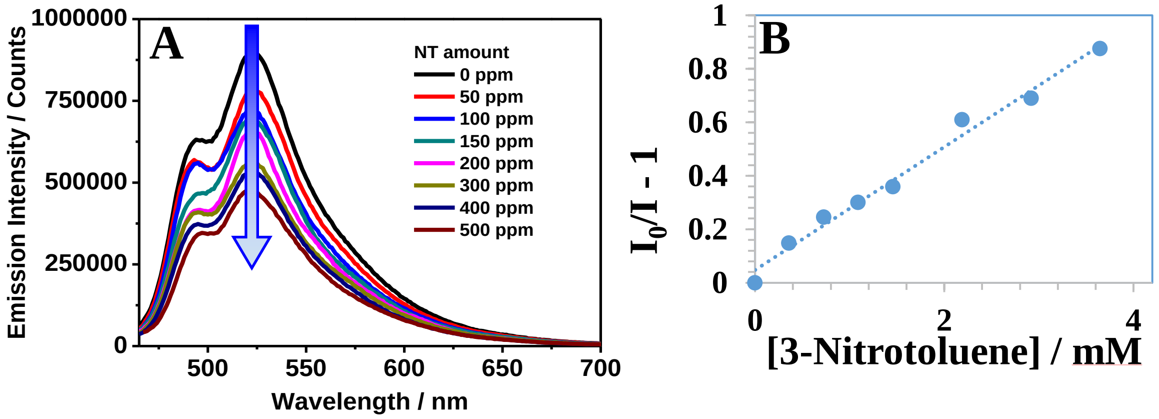

Figure S23. (A) Emission spectra of Ce-CAU-24-TBAPy in acetone without and in presence of different amounts (0, 50, 100, 150, 200, 300, 400 and 500 ppm) of 3-Nitrotoluene (NT). The excitation wavelength was 450 nm. (B) Stern-Volmer analysis of the emission quenching upon addition of NT. $K_S = 2.31 \times 10^2\ M^{-1}$.

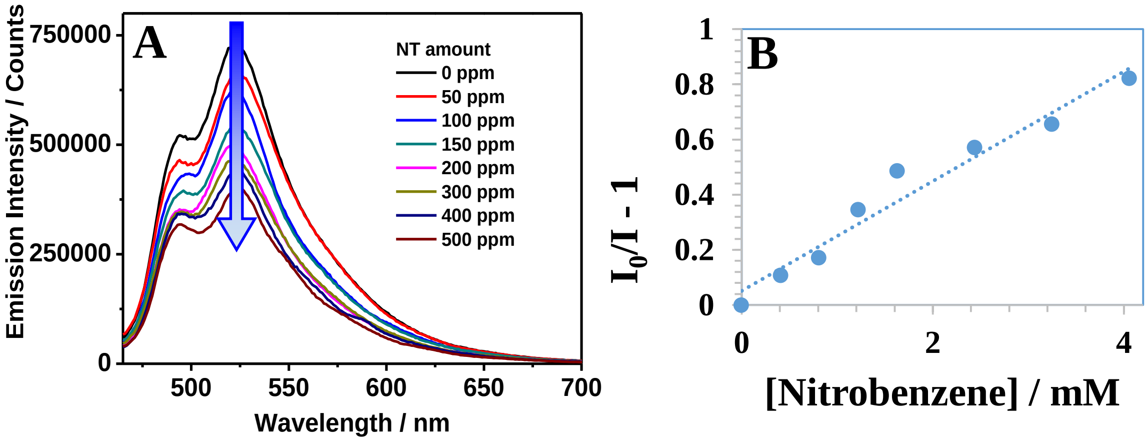

Figure S24. (A) Emission spectra of Ce-CAU-24-TBAPy in acetone without and in presence of different amounts (0, 50, 100, 150, 200, 300, 400 and 500 ppm) of Nitrobenzene (NB). The excitation wavelength was 450 nm. (B) Stern-Volmer analysis of the emission quenching upon addition of NB. $K_S = 1.99 \times 10^2\ M^{-1}$.

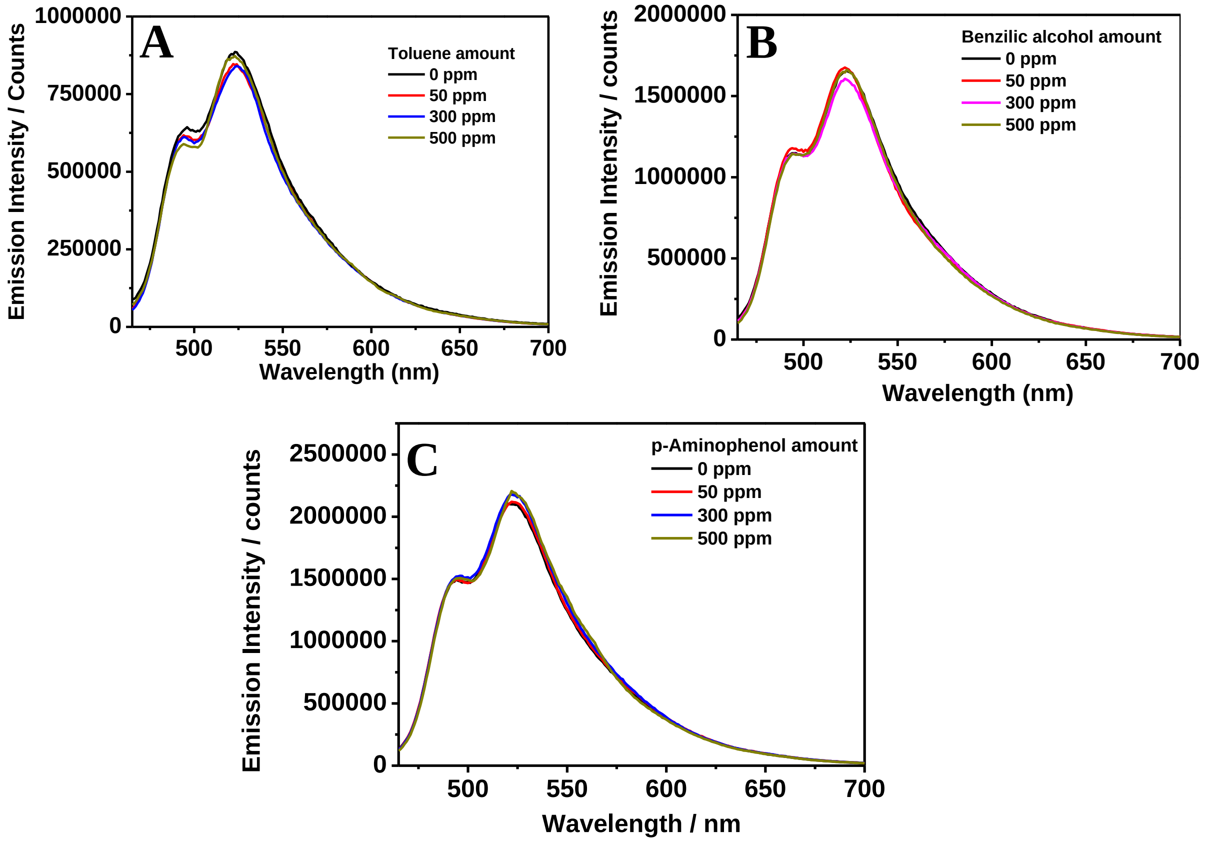

Figure S25. Emission spectra of Ce-CAU-24-TBAPy in acetone without and in presence of different amounts (0, 50, 300 and 500 ppm) of (A) toluene, (B) benzylic alcohol and (C) p-aminophenol. Excitation wavelength: 450 nm.

Table S1. Values of the time constants (τ_i) and normalized (to 100) pre-exponential factors (a_i) and contributions (c_i) obtained from the best multiexponential fits of the emission decays of Ce-NU-1000 in acetone, upon excitation at 370 nm.

$\lambda_{Obs/nm}$	τ_1/ns (±0.10)	a_1	c_1	τ_2/ns (±0.20)	a_2	c_2	τ_3/ns (±0.20)	a_3	c_3
450	0.18	51	10	1.75	49	90	14.3	-	-
475		47	3		39	23		14	74
500		39	1		32	12		29	87
525		40	1		20	6		40	93
550		37	1		20	5		43	94
575		36	1		19	4		45	95

Table S2. Values of the time constants (τ_i) and normalized (to 100) pre-exponential factors (a_i) and contributions (c_i) obtained from the best multiexponential fits of the magic-angle emission decays of Ce-CAU-24-TBAPy upon excitation at 370 nm.

$\lambda_{Obs/nm}$	τ_1/ns (±0.10)	a_1	c_1	τ_2/ns (±0.20)	a_2	c_2	τ_3/ns (±0.20)	a_3	c_3
475	0.19	66	16	1.64	31	64	5.28	3	20
500		36	5		57	68		7	27
525		27	3		63	64		10	33
550		23	2		61	52		16	46
575		19	2		58	42		23	56
600		13	1		46	26		41	73
625		-	-		38	16		62	84
650		-	-		7	2		93	98

Table S3. Values of the time constants (τ_i) and normalized (to 100) pre-exponential factors (a_i) and contributions (c_i) obtained from the best multiexponential fits of the emission decays of Ce-CAU-24-TBAPy in acetone upon excitation at 433 and 370 nm.

λ_{Exc}/nm	λ_{Obs}/nm	τ_1/ns (±0.10)	a_1	c_1	τ_2/ns (±0.20)	a_2	c_2	τ_3/ns (±0.20)	a_3	c_3
**433**	475	0.21	32	10	1.45	68	90	5.31	-	-
	500		26	5		72	86		2	9
	550		9	1		85	65		6	35
	600		-	-		72	34		28	66
**370**	475	0.19	66	16	1.64	31	64	5.28	3	20
	500		36	5		57	68		7	27
	550		23	2		61	52		16	46
	600		13	1		46	26		41	73

Table S4. Values of the time constants (τ_i) and pre-exponential factors (a_i) normalized (to 100) obtained from a multiexponential fit of the emission decays of Ce-CAU-24-TBAPy upon excitation at 370 nm under the fluorescence microscope at the indicated points (1, 2 and 3) and regions (DI and DII) (Figures 3A and 3B).

Point / Region	τ_1 / ns (±0.10)	a_1	τ_2 / ns (±0.50)	a_2	τ_3 / ns (±0.50)	a_3
**1**	0.49	69	2.60	24	8.80	7
**2**	0.52	78	2.60	17	8.60	5
**3**	0.48	83	2.40	13	8.10	4
**DI**	0.53	90	2.40	10	-	-
**DII**	-	-	2.50	60	8.81	40

Table S5. Values of the time constants (τ_i) and normalized (to 100) pre-exponential factors (a_i) obtained from the best multiexponential fits of the emission decays of Ce-CAU-24-TBAPy single crystals (A to F)) under the fluorescence microscope upon excitation at 370 nm, and collection at different points on each crystal (as shown in Figure S8, crystals A to F).

Crystal A:

Point	τ_1 / ns (±0.10)	a_1	τ_2 / ns (±0.50)	a_2	τ_3 / ns (±0.50)	a_3
1	0.47	69	2.30	24	7.50	7
2	0.49	73	1.99	16	7.45	11
3	0.52	77	2.37	14	7.60	9
4	0.51	78	2.01	15	7.71	7
5	0.47	73	2.30	17	7.80	10

Crystal B:

Point	τ_1 / ns (±0.10)	a_1	τ_2 / ns (±0.50)	a_2	τ_3 / ns (±0.50)	a_3
1	0.51	81	2.33	14	7.81	5
2	0.57	83	2.10	13	8.30	4
3	0.58	82	2.30	14	8.00	4
4	0.57	82	2.20	13	7.90	5
5	0.53	86	2.20	11	7.60	3
6	0.54	82	2.40	13	8.70	5

Crystal C:

Point	τ_1 / ns (±0.10)	a_1	τ_2 / ns (±0.50)	a_2	τ_3 / ns (±0.50)	a_3
1	0.62	78	2.27	14	8.10	8
2	0.63	79	2.40	18	7.90	3
3	0.65	82	2.56	13	8.50	2
4	0.63	79	2.30	13	8.00	8
5	0.58	71	1.85	19	8.30	10

Crystal D:

Point	τ_1 / ns (±0.10)	a_1	τ_2 / ns (±0.50)	a_2	τ_3 / ns (±0.50)	a_3
1	0.49	69	2.60	24	8.80	7
2	0.52	78	2.60	17	8.60	5
3	0.48	83	2.40	13	8.10	4

Crystal E:

Point	τ_1 / ns (±0.10)	a_1	τ_2 / ns (±0.50)	a_2	τ_3 / ns (±0.50)	a_3
1	0.43	79	2.10	16	7.80	5
2	0.51	69	2.65	14	8.01	7
3	0.59	78	2.30	15	7.90	7

Crystal F:

Point	τ_1 / ns (±0.10)	a_1	τ_2 / ns (±0.50)	a_2	τ_3 / ns (±0.50)	a_3
1	0.49	63	2.52	29	8.40	8
2	0.36	71	2.40	21	8.60	8
3	0.18	98	1.20	1	6.20	1
4	0.39	72	1.98	22	7.90	6
5	0.53	68	2.70	24	8.71	8
6	0.54	65	2.85	27	8.82	8

Table S6. Values of time constants (τ_i) and normalized (to 100) pre-exponential factors (a_i) obtained from the fit of the transient absorption decays of the indicated samples, upon excitation at 430 nm and observation as indicated. The negative value of a_i reflects that the signal is negative.

Sample	$\lambda_{Obs/nm}$	τ_1/μs (±0.05)	a_1	τ_2/μs (±0.20)	a_2	τ_3/μs (±0.20)	a_3
H_4TBAPy	450	0.56	-	2.15	100		
	500		42		58		
	550		100		-		
	650		100		-		
Ce-NU-1000	450	0.64	-79	4.91	-21		
	500		-77		-23		
	550		-74		-26		
	575		-73		-27		
Ce-CAU-24-TBAPy	500	0.10	100	1.59	-91	13.43	-9
	550		100		-82		-18
	600		100		-80		-20
	650		100		-77		-23

Table S7. Values of the time constants (τ_i) and normalized (to 100) pre-exponential factors (a_i) obtained from a multiexponential fit of the femtosecond emission transients of Ce-CAU-24-TBAPy in acetone upon excitation at 370 nm, and observation as indicated. The negative sign for a_i indicates a rising component in the emission signal.

$\lambda_{Obs/nm}$	τ_1/fs (±50)	a_1	τ_2/ps (±50)	a_2
440	154	100	-	-
460	141	100	-	-
480	164	100	-	-
510	188	73	245	27
530	179	60	213	40
550	150	-100	209	100
570	165	-100	235	100

Table S8. Spectral region of the absorbing ICT (intramolecular charge transfer), twisting, excimers and LCCT (ligand-to-cluster charge transfer) structures of the examined systems in acetone solutions upon fs-excitation. Those between brackets are relatively weak populated states.

	**Visible Region**		**NIR Region**	
	**600 nm**	**700 nm**	**900 nm**	**1000 nm**
**H_4TBAPy**	**(ICT)**	**ICT**	**ICT<br>Twist**	**ICT<br>Twist**
**Ce-NU-1000**	**LCCT**	**ICT<br>Excimer**	**LCCT<br>(ICT)**	**(LCCT)<br>(ICT)**
**Ce-CAU-24-TBAPy**	**LCCT**	**ICT**	**LCCT<br>(ICT)**	**LCCT<br>ICT**

Table S9. Fitting parameters of the emission decays of Ce-CAU-24-TBAPy in acetone with and without 500 ppm of TNB and TNP, upon excitation at 370 nm and observation at 475 and 600 nm.

Nitroaromatic	Amount/ ppm x 3 mol	$\lambda_{Obs/nm}$	τ_1/ns (±0.10)	a_1	c_1	τ_2/ns (±0.20)	a_2	c_2	τ_3/ns (±0.20)	a_3	c_3
TNB	0 ppm	475	0.21	57	18	1.61	35	68	5.65	8	14
		600		15	4		50	30		35	66
	500 ppm	475	0.20	38	9	1.26	61	82	5.36	1	9
		600		13	1		63	36		24	63
TNP	0 ppm	475	0.21	57	18	1.61	35	68	5.65	8	14
		600		15	4		50	30		35	66
	500 ppm	475	0.31	56	18	1.65	37	66	5.71	6	12
		600		11	3		52	31		37	66

References:

1. Organero, J. A.; Tormo, L.; Douhal, A., Caging ultrafast proton transfer and twisting motion of 1-hydroxy-2-acetonaphthone. *Chem. Phys. Lett.* **2002,** *363* (3), 409-414.
2. Randino, C.; Ziółek, M.; Gelabert, R.; Organero, J. A.; Gil, M.; Moreno, M.; Lluch, J. M.; Douhal, A., Photo-deactivation pathways of a double H-bonded photochromic Schiff base investigated by combined theoretical calculations and experimental time-resolved studies. *Phys. Chem. Chem. Phys.* **2011,** *13* (33), 14960-14972.
3. Alarcos, N.; Cohen, B.; Douhal, A., Photodynamics of a Proton-Transfer Dye in Solutions and Confined Within NaX and NaY Zeolites. *J. Phys. Chem. C* **2014,** *118* (33), 19431-19443.
4. Gil, M.; Douhal, A., Femtosecond dynamics of a non-steroidal anti-inflammatory drug (piroxicam) in solution: The involvement of twisting motion. *Chem. Phys.* **2008,** *350* (1), 179-185.
